# Role reversal of functional identity in host factors: Dissecting features affecting pro-viral versus antiviral functions of cellular DEAD-box helicases in tombusvirus replication

**DOI:** 10.1371/journal.ppat.1008990

**Published:** 2020-10-09

**Authors:** Cheng-Yu Wu, Peter D. Nagy

**Affiliations:** Department of Plant Pathology, University of Kentucky, Lexington, Lexington, United States of America; Agriculture and Agri-Food Canada, CANADA

## Abstract

Positive-stranded (+)RNA viruses greatly exploit host cells to support viral replication. However, unlike many other pathogens, (+)RNA viruses code for only a limited number of genes, making them highly dependent on numerous co-opted host factors for supporting viral replication and other viral processes during their infections. This excessive dependence on subverted host factors, however, renders (+)RNA viruses vulnerable to host restriction factors that could block virus replication. Interestingly, cellular ATP-dependent DEAD-box RNA helicases could promote or inhibit the replication of *Tomato bushy stunt virus* (TBSV) replication. However, it is currently unknown what features make a particular DEAD-box helicase either pro-viral or antiviral. In this work, we succeeded in reversing the viral function of the antiviral DDX17-like RH30 DEAD-box helicase by converting it to a pro-viral helicase. We also turned the pro-viral DDX3-like RH20 helicase into an antiviral helicase through deletion of a unique N-terminal domain. We demonstrate that in the absence of the N-terminal domain, the core helicase domain becomes unhinged, showing altered specificity in unwinding viral RNA duplexes containing *cis*-acting replication elements. The discovery of the sequence plasticity of DEAD-box helicases that can alter recognition of different *cis*-acting RNA elements in the viral genome illustrates the evolutionary potential of RNA helicases in the arms race between viruses and their hosts, including key roles of RNA helicases in plant innate immunity. Overall, these findings open up the possibility to turn the pro-viral host factors into antiviral factors, thus increasing the potential antiviral arsenal of the host for the benefit of agriculture and health science.

## Author summary

The largest group of eukaryotic viruses, the positive-strand RNA viruses, depends greatly on co-opting host components to support their replication. This dependence on host factors by these viruses also makes them vulnerable to antiviral factors. This is well-illustrated in case of tombusviruses, a small RNA viruses of plants. Tombusviruses co-opt many host factors to support various steps in their replication. Among these host factors are cellular DEAD-box helicases, which help remodeling viral RNA structures during the RNA replication process. However, similar cellular helicases remodel the viral RNAs incorrectly, making them antiviral or restriction factors. To gain insights into what makes a particular DEAD-box helicase pro-viral or antiviral, in this work, we converted the antiviral plant RH30 helicase into a pro-viral helicase through modifying the N-terminal sequences. We also succeeded to turn the originally pro-viral plant RH20 helicase into an antiviral helicase using a similar strategy. By characterizing the newly acquired functions of these helicases, we obtained valuable insights into what features make these helicases either pro-viral or antiviral. These discoveries have implications to better understand the arms race between viruses and hosts. In addition, it opens up the opportunity to generate new antiviral tools by converting pro-viral host factors into antiviral factors, thus enhancing our molecular tools against the ever-evolving RNA viruses.

## Introduction

Positive-stranded (+)RNA viruses exploit host cells by co-opting many cellular factors to facilitate viral replication. In addition, many (+)RNA viruses take advantage of metabolic pathways, subcellular membranes and intracellular trafficking to build elaborate membranous viral replication compartments (also called viral replication organelles, VROs), which are the sites of viral replication, in the cytosol of the infected cells [1–8]. The emerging picture is that the co-opted host factors affect many steps of RNA virus replication. For example, the assembly of membrane-bound viral replicase complexes (VRCs) is assisted by host translation initiation and elongation factors, protein chaperones, RNA-modifying enzymes, SNARE and ESCRT proteins, the actin network, and lipids [1–3,7,9,10].

The mechanistic roles of host factors in viral RNA replication is intensively studied with *Tomato bushy stunt virus* (TBSV) and other tombusviruses infecting plants. [7,8,11]. Expression of the two TBSV replication proteins, termed p33 and p92^pol^, and a replicon (rep)RNA leads to efficient viral replication in yeast (*Saccharomyces cerevisiae*) surrogate host [9,12,13]. The abundant p33, which overlaps with the N-terminal region of p92^pol^, is an RNA chaperone and a master regulator of replication, whereas p92^pol^ is the RdRp [7]. Several *cis*-acting replication elements have been defined in the TBSV (+) and (-) RNAs, which have critical functions in RNA template selection, RNA recruitment, in the assembly of VRCs, activation of the p92 RdRp and in viral RNA synthesis [13–19].

Among the largest family of cellular proteins affecting TBSV replication is the ATP-dependent DEAD-box RNA helicases. Multiple global screens in yeast with TBSV have identified 11 yeast helicases (10 DEAD-box and 1 DEAH-box helicases), which are involved in viral replication or viral RNA recombination [20–26]. Moreover, TBSV, which lacks its own helicase, usurps several plant ATP-dependent DEAD-box RNA helicases to promote TBSV RNA replication. For example, the plant DDX3-like RH20, DDX5-like RH5 and the eIF4AIII-like RH2 DEAD-box proteins affect plus-strand synthesis, viral genome integrity and viral RNA recombination [27–30]. On the other hand, the antiviral DDX17-like RH30 DEAD-box protein is re-localized from the nucleus to the site of tombusvirus replication via interacting with the TBSV p33 and p92^pol^ replication proteins. RH30 inhibits tombusvirus replication through blocking several steps in the replication process. The action of RH30 DEAD-box helicase interferes with VRC assembly, viral RdRp activation and the specific interaction between p33 replication protein and the viral (+)RNA [31].

DEAD-box helicases are a large family of RNA helicases and they are involved in cellular metabolism [32–35], and in responses to abiotic stress and pathogen infections [36–41]. DEAD-box helicases unwind RNA duplexes, affect RNA folding and remodel RNA-protein complexes [42]. Interestingly, DEAD-box helicases can open up completely double-stranded RNA regions by directly loading on duplexes and opening up a limited number of base pairs. This unwinding mode is termed local strand separation [34,42]. Ever increasing data suggest that cellular DEAD-box helicases affect translation and replication of many RNA viruses [43–46]. For example, turnip mosaic virus and brome mosaic virus co-opt cellular DEAD-box helicases for pro-viral functions in translation or replication [44,47,48]. HIV infections are affected by several helicases providing either pro-viral or antiviral functions [49,50]. Thus, the emerging idea is that cellular DEAD-box helicases are important co-opted host factors for several viruses, whereas other cellular DEAD-box helicases play active roles in restricting RNA virus replication. Overall, cellular DEAD-box helicases perform critical roles in virus-host interactions.

In this work, we characterized domains that specify the viral restriction function of the cellular DDX17-like RH30 DEAD-box RNA helicase and the pro-viral function of the DDX3-like RH20 helicase in tombusvirus replication. Expression of deletion mutants of RH30 and RH20 in *Nicotiana benthamiana* plants revealed that the unique N-terminal domain is important in determining the viral functions of these helicases. To gain insights into what makes a particular DEAD-box helicase either pro-viral or antiviral, we converted the antiviral plant RH30 helicase into a pro-viral helicase through modifying the N-terminal sequences. We also turned the originally pro-viral plant RH20 helicase into an antiviral helicase using a similar strategy. We found that the antiviral helicases target a *cis*-acting element (namely RII(+)-SL) in the viral (+)RNA, which is critical during the early steps of replication. Therefore, we suggest that the antiviral DEAD-box helicase must act at the earliest steps in the replication process to inhibit TBSV replication. On the contrary, the pro-viral helicases in this work targeted a different *cis*-acting element (namely RI that includes the 5’ noncoding region) in the viral RNA, which is needed for (+)RNA synthesis, a latter step in the replication process. This surprising outcome suggests that the antiviral or pro-viral roles of host DEAD-box helicases could be reversed when regulatory/specifying domains are removed from or exchanged between the helicase proteins. These findings on cellular DEAD-box helicases could have major implications in the arms race between the virus and plant hosts to influence the outcome of viral infections.

## Results

### Dissecting domains specifying the viral restriction function of the cellular RH30 DEAD-box RNA helicase in tombusvirus replication

**Fig 1:**
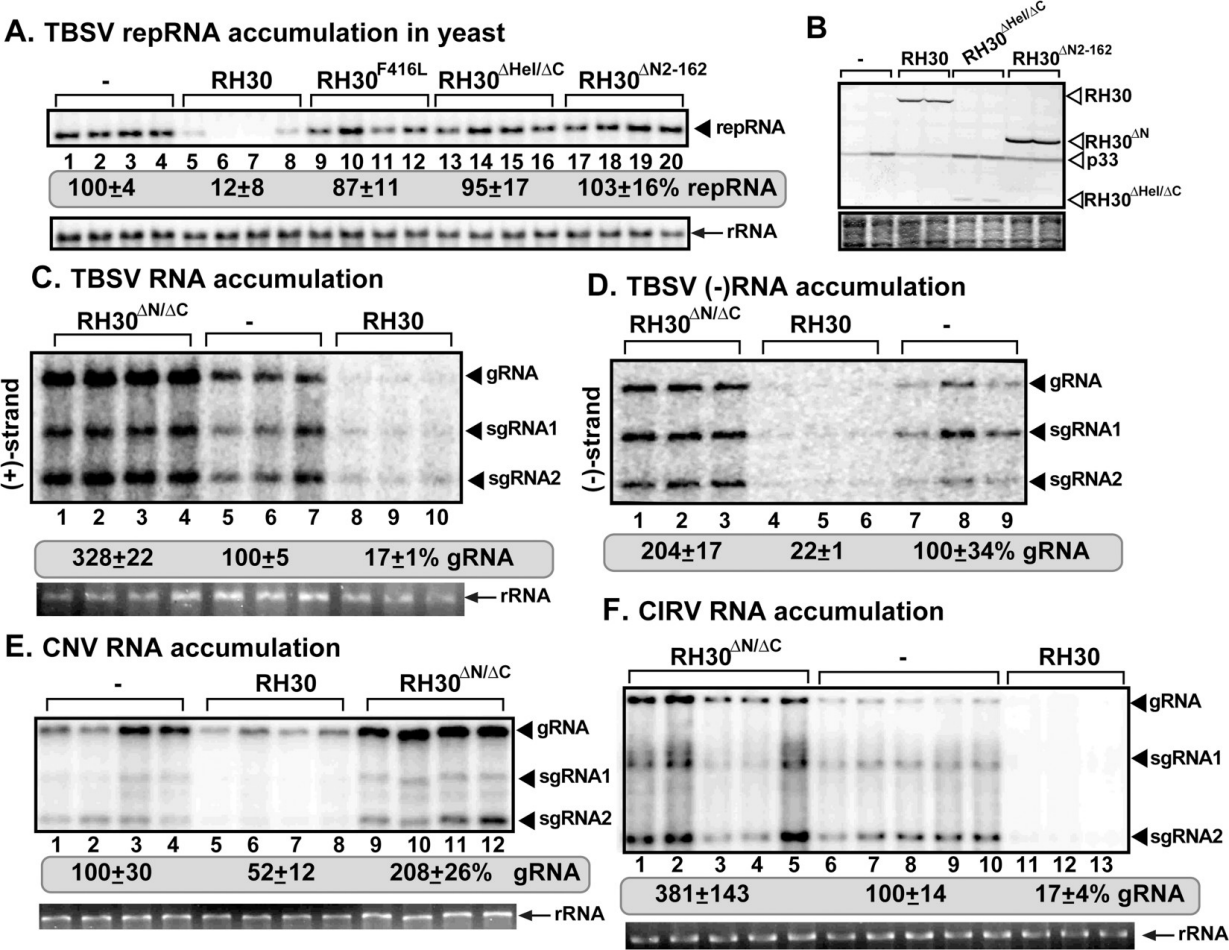
Effects of expression of truncation mutants of the antiviral RH30 DEAD-box helicase on tombusvirus genomic (g)RNA replication in *N*. *benthamiana* plant and in yeast surrogate host. (A) The effect of expression of truncation mutants of RH30 on TBSV replication in yeast. Top panel: Northern blot analysis of TBSV repRNA using a 3’ end specific probe shows the level of accumulation of repRNA in wt yeast strain expressing truncation mutants of RH30. Viral proteins His_6_-p33 and His_6_-p92^pol^ were expressed from plasmids from the *CUP1* promoter, while DI-72(+) repRNA was expressed from the *GAL1* promoter. His_6_-RH30 derivatives were expressed from a plasmid. Northern blots were evaluated with a phosphoimager using Imagequant software. Standard error was calculated based on three repeats. Bottom panel: Northern blot with 18S ribosomal RNA specific probe was used as a loading control. (B) Western blot analysis of the level of His_6_-p33, and His_6_-RH30 with anti-His antibody. The samples were from wt yeast strain expressing RH30 derivatives. (C) *N*. *benthamiana* plants expressing RH30^ΔN/ΔC^ helicase were inoculated with (C-D) TBSV, (E) CNV, (F) CIRV, respectively. Top panel: Northern blot analyses of tombusvirus gRNA using a 3’ end specific probe shows increased accumulation of gRNA and subgenomic RNAs in plants expressing RH30^ΔN/ΔC^ helicase when compared with plants expressing RH30 or in control plants (treatment with the empty vector). Bottom panel: Ethidium-bromide stained gel shows 18S ribosomal RNA as a loading control. Note that the northern blot analyses of TBSV (+)gRNA is shown in panel C and (-)gRNA using a 3’ end specific probe for (-)RNA detection is shown in panel D. Each experiment was repeated at least three times.

Sequence comparison of the DDX17-like RH30 DEAD-box RNA helicase from *Arabidopsis*, which shows strong restriction function against tombusviruses [31], with the pro-viral DDX3-like RH20 DEAD-box RNA helicase [51] suggests the existence of three domains. The highly conserved core DEAD-box helicase domain is present in the center of these two helicases, whereas the N-terminal and short C-terminal domains are more divergent from one another (S1 Fig). To identify what domains are responsible for the antiviral function of RH30 DEAD-box RNA helicase, we expressed deletion mutants of RH30 helicase in *N*. *benthamiana* plants via agroinfiltration. Interestingly, similar to the helicase functional mutant RH30^F416L^, the N-domain deletion mutant of RH30 also lost its antiviral effect against TBSV replication in the inoculated leaves (RH30^ΔN2-162^, S1 Table, S2 Fig). Similarly, expression of the N-terminal domain deletion mutant RH30^ΔN2-162^ did not show antiviral activity against TBSV replicon (rep)RNA in yeast surrogate host (Fig 1A, lanes 17–20 versus 5–8). Replication of another tombusvirus, carnation Italian ringspot virus (CIRV), which utilizes the outer membranes of mitochondria to build VROs, was also not inhibited in *N*. *benthamiana* by the transient expression of the RH30^ΔN2-162^ missing the N-terminal domain (S1 Table). Taken together, we suggest that RH30 requires the unique N-terminal domain to be an active restriction factor against TBSV and CIRV replication.

As expected, deletion of the core DEAD-box helicase domain in RH30 nullified its antiviral activity against both TBSV and CIRV in *N*. *benthamiana* (RH30^ΔHel^, S1 Table). In contrast, deletion of the short C-terminal domain in RH30 did not interfere with the restriction function in *N*. *benthamiana* (RH30^ΔC547-592^, S1 Table). Combined deletion of the core DEAD-box helicase and C-terminal domains in RH30 nullified the antiviral activity of RH30 in *N*. *benthamiana* (RH30^ΔHel/ΔC^, S1 Table) and in yeast (Fig 1A, lanes 13–16). However, combined deletion of both N- and C-terminal domains, which left only the core DEAD-box helicase domain in RH30, converted this helicase into a pro-viral factor by increasing TBSV replication by ~2-to-3-fold in *N*. *benthamiana* (RH30^ΔN/ΔC^, S1 Table and Fig 1C, lanes 1–4). Expression of RH30^ΔN/ΔC^, also enhanced the accumulation of the closely-related cucumber necrosis virus (CNV) and CIRV accumulation by 2-to-3-fold in N. *benthamiana* (Fig 1E and 1F). This surprising outcome suggests that an antiviral DEAD-box helicase could become a pro-viral factor when inhibitory (or regulatory) domains are removed from the protein.

### The pro-viral RH30^ΔN/ΔC^ DEAD-box helicase is re-localized into the tombusvirus replication compartment in plants

**Fig 2:**
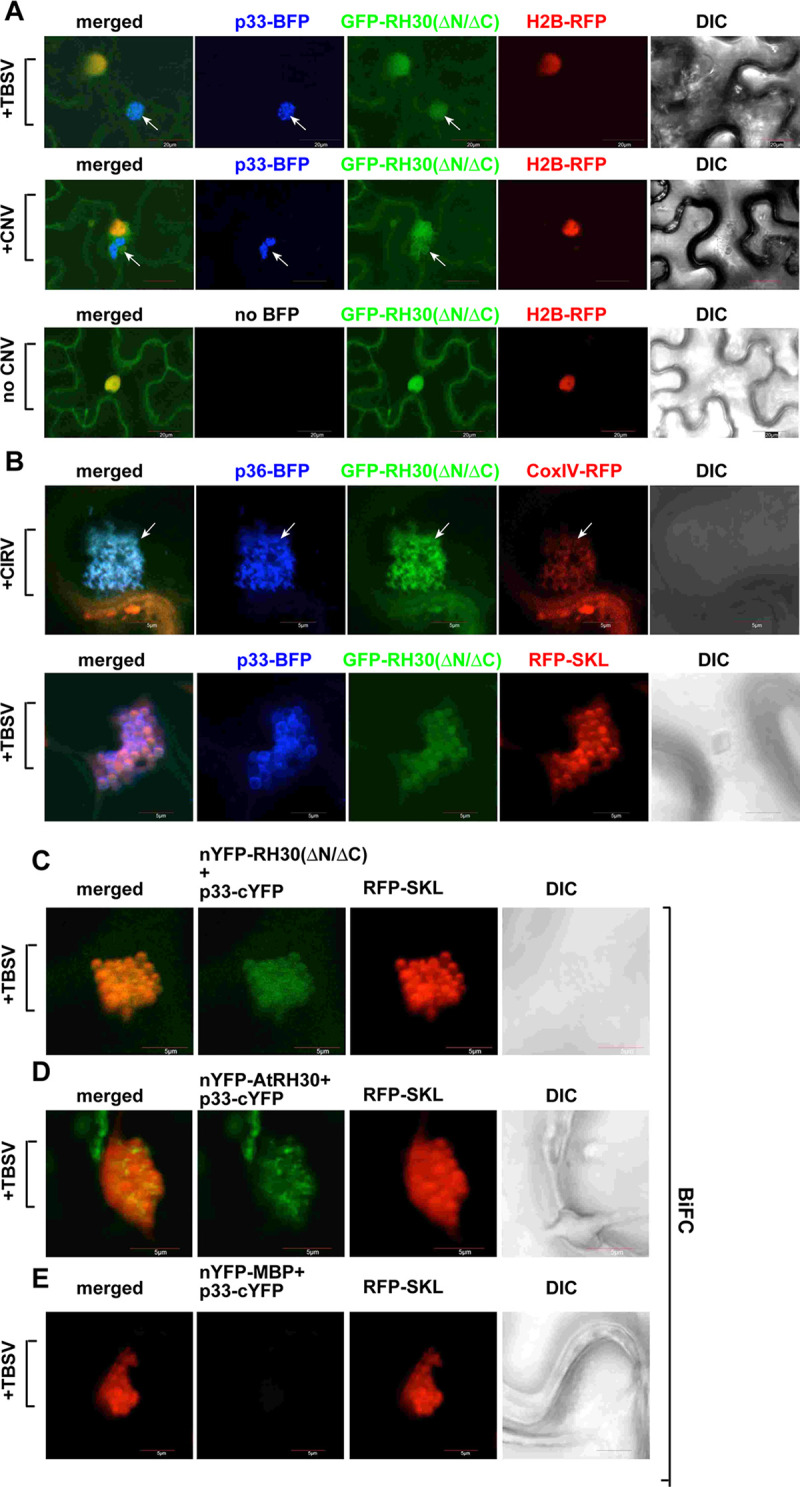
Confocal microscopy shows the retargeting of the nuclear/cytosolic RH30^ΔN/ΔC^ helicase into the large replication compartment in whole plants infected with a tombusvirus. (A) Confocal microscopy images show that most of RH30^ΔN/ΔC^ helicase is re-targeted into the replication compartment marked by the BFP-tagged p33 replication protein (pointed by arrows) in *N*. *benthamiana* plants infected with either TBSV or CNV. Bottom panel: distribution of RH30^ΔN/ΔC^ helicase in the absence of viral components. The nucleus is marked by a histone protein (transgenic plants expressing nucleus marker RFP-H2B). Scale bars represent 20 μm. (B) Confocal microscopy images show that the re-targeted GFP-RH30^ΔN/ΔC^ helicase is present in the VRO, marked by the CIRV p36-BFP replication protein and CoxIV-RFP mitochondrial marker. Arrows point at the large VRO. Expression of the above proteins from the 35S promoter was done after co-agroinfiltration into *N*. *benthamiana* leaves. The agro-infiltrated leaves were collected for confocal imaging at 2.5 days post agro-infiltration. Bottom panel: The TBSV p33-BFP replication protein and RFP-SKL peroxisomal matrix marker co-localize with the GFP-RH30^ΔN/ΔC^ helicase within the large VRO. Scale bars represent 5 μm. Each experiment was repeated twice. (C) Interactions between TBSV p33 replication protein and the RH30^ΔN/ΔC^ helicase were detected by BiFC. The TBSV p33-cYFP replication protein and the nYFP-RH30^ΔN/ΔC^ and the RFP-SKL peroxisomal marker protein were expressed via agroinfiltration. The merged image shows the efficient co-localization of the peroxisomal RFP-SKL with the BiFC signals, indicating that the interaction between the tombusvirus replication protein and the recruited RH30^ΔN/ΔC^ helicase takes place in VROs, which consist of aggregated peroxisomes. Scale bars represent 5 μm. (D) Interactions between TBSV p33 replication protein and the RH30 helicase were detected by BiFC. See further details in panel C. (E) Control BIFC experiment to show no fluorescent signals are generated in the absence of interaction. See further details in panel C.

The antiviral RH30 DEAD-box helicase has to re-localize into the large p33-containing replication compartment in order to restrict tombusvirus replication [31]. To test if the pro-viral RH30^ΔN/ΔC^ helicase is also present in VROs, we co-expressed GFP-tagged RH30^ΔN/ΔC^ with p33-BFP replication protein and either H2B-RFP (nuclear marker) or RFP-SKL (a peroxisomal matrix marker) in *N*. *bentamiana* infected with TBSV or CNV (Fig 2A and 2B). Confocal microscopy analysis of *N*. *bentamiana* leaves showed the partial re-localization of GFP-RH30^ΔN/ΔC^ into the large TBSV or CNV VROs, which consist of aggregated peroxisomes. GFP-RH30^ΔN/ΔC^ helicase was also partially re-targeted in CIRV-infected *N*. *benthamiana* cells into the CIRV p36 and p95^pol^-containing VROs (Fig 2B, top panel), which consist of aggregated mitochondria decorated by CoxIV-RFP marker [52,53]. Based on these experiments, we propose that the nuclear and cytosolic-localized pro-viral RH30^ΔN/ΔC^ helicase is re-targeted efficiently into the tombusvirus replication compartment, similar to the full-length RH30 helicase with antiviral function.

We also analyzed the subcellular distribution of GFP-RH30^ΔN(ΔN2-162)^, which showed no significant antiviral activity when expressed in *N*. *bentamiana* leaves (Fig 1A, lanes 17–20, and S1 Table). In the absence of tombusviral infection, GFP-RH30^ΔN^ showed mostly nuclear localization in *N*. *bentamiana* leaves or in protoplasts (S3 Fig). This pattern of localization is different from the nuclear and cytosolic-localization pattern of the anti-viral RH30 or the pro-viral RH30^ΔN/ΔC^ helicases (S3 Fig). The partial redistribution of GFP-RH30^ΔN^ from nucleus to the VROs was detectable in TBSV-infected plants, but it was somewhat inefficient (S3 Fig). This unusually strong nuclear localization of GFP-RH30^ΔN^ might explain its lack of activity during TBSV replication. Accordingly, we have shown that the antiviral activity of the full-length RH30 depended on its efficient redistribution from the nucleus to the VROs [31]. Future detailed experiments are needed to determine the factors affecting the subcellular localization of these cellular helicases and mutants.

To test if the RH30^ΔN/ΔC^ helicase interacts with the tombusvirus replication protein, we have conducted bimolecular fluorescence complementation (BiFC) experiments in *N*. *benthamiana* leaves. Comparison with the antiviral full-length RH30 helicase, RH30^ΔN/ΔC^ helicase also showed interaction with the TBSV p33 replication protein within the viral replication compartment, marked by the peroxisomal matrix marker RFP-SKL (Fig 2C–2E).

### The pro-viral RH30^ΔN/ΔC^ DEAD-box helicase promotes tombusvirus RNA synthesis *in vitro*

**Fig 3:**
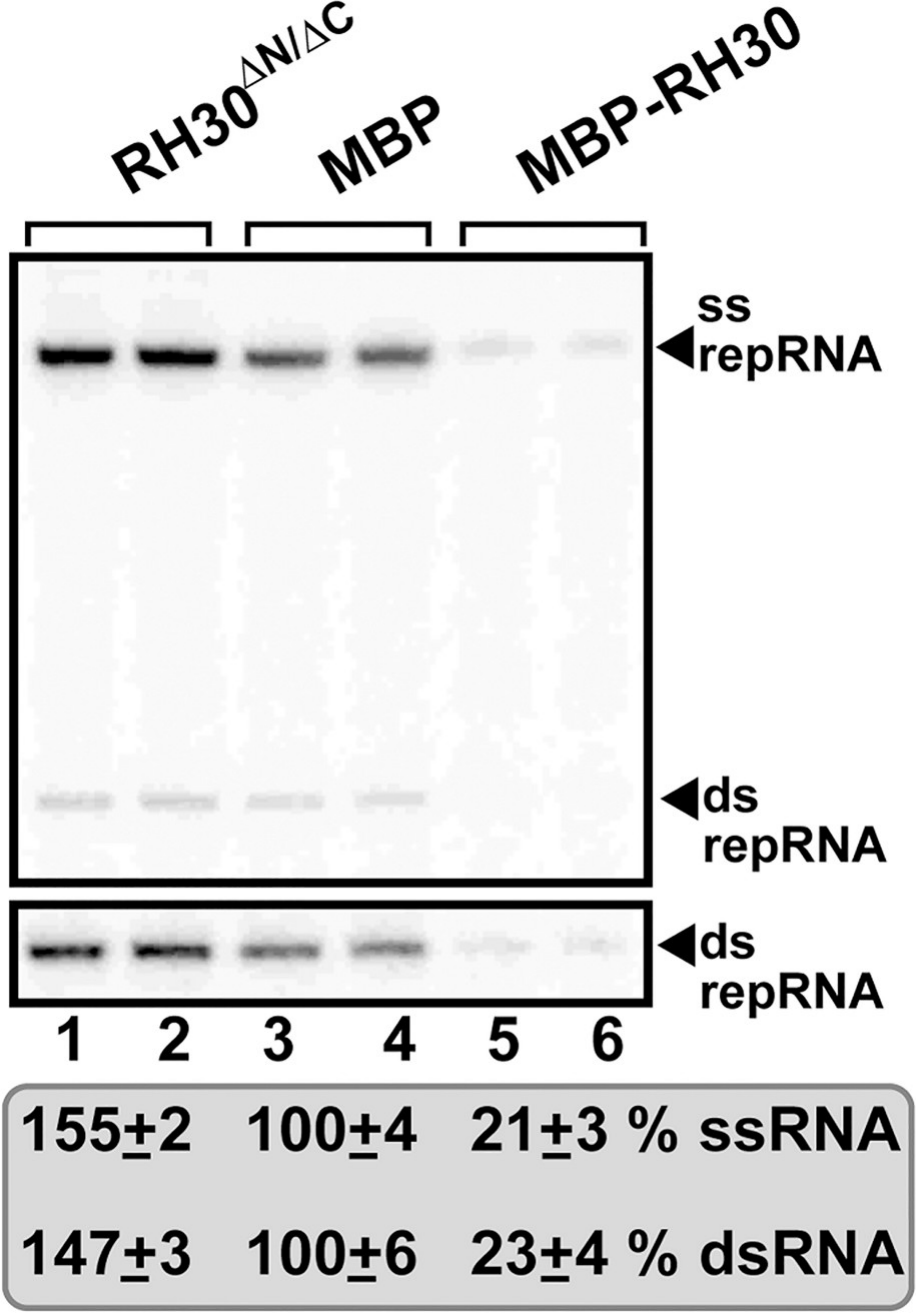
Enhanced TBSV repRNA accumulation by RH30^ΔN/ΔC^ helicase in an *in vitro* replicase reconstitution assay based on CFE obtained from wt yeast. The purified recombinant tombusvirus p33 and p92^pol^ replication proteins from *E*. *coli* were added in combination with the template (+)repRNA to program the yeast CFE preparation in vitro. The affinity-purified recombinant MBP-RH30^ΔN/ΔC^ (1.9 μM), MBP-RH30 (1.9 μM) or MBP (1.9 μM), as a control, were added to the reactions. Non-denaturing PAGE shows the accumulation of ^32^P-labeled (+)repRNA and the dsRNA replication intermediate products made by the reconstituted TBSV replicases. The bottom image shows the enhanced image of the dsRNA products in the top image. Each experiment was repeated three times.

To gain deeper insights into the acquired pro-viral function of RH30^ΔN/ΔC^ helicase, we affinity-purified the recombinant RH30^ΔN/ΔC^ helicase from *E*. *coli*, followed by testing its activity *in vitro* in a TBSV replicase reconstitution assay with purified recombinant TBSV p33 and p92^pol^ replication proteins [18,54]. Addition of the purified RH30^ΔN/ΔC^ helicase to the replicase reconstitution assay, which is based on yeast cell-free extract (CFE), led to increased TBSV repRNA replication by ~50% (Fig 3, lanes 1–2). The double-stranded repRNA replication intermediate was also produced by ~40% more efficiently in the presence of RH30^ΔN/ΔC^ helicase. These *in vitro* data suggest that RH30^ΔN/ΔC^ helicase likely promotes RNA synthesis or the VRC assembly during TBSV replication. This idea is also supported by the increased level of both (-)RNAs and (+)RNAs observed when RH30^ΔN/ΔC^ helicase was expressed in *N*. *benthamiana* (Fig 1C, lanes 1–4 and 1D, lanes 1–3). This is in contrast with the strong inhibitory effect of the full-length RH30 DEAD-box helicase on TBSV RNA replication (Fig 3, lanes 5–6) [31]. Please note that the pro-viral activity of RH30^ΔN/ΔC^ helicase is not fully identical with that of the previously shown DDX3-like yeast Ded1 or the plant RH20 helicases, which mostly enhanced (+)RNA synthesis [29]. The enhanced activity of RH30^ΔN/ΔC^ helicase in (-)-strand synthesis is similar to some Ded1 mutants [27] and it is likely due to a yet undefined helicase function during VRC assembly or the use of (+)RNA template during (-)RNA synthesis.

### The altered specificity of pro-viral RH30^ΔN/ΔC^ DEAD-box helicase in unwinding critical cis-acting elements in the viral RNA in comparison with the antiviral RH30 helicase

**Fig 4:**
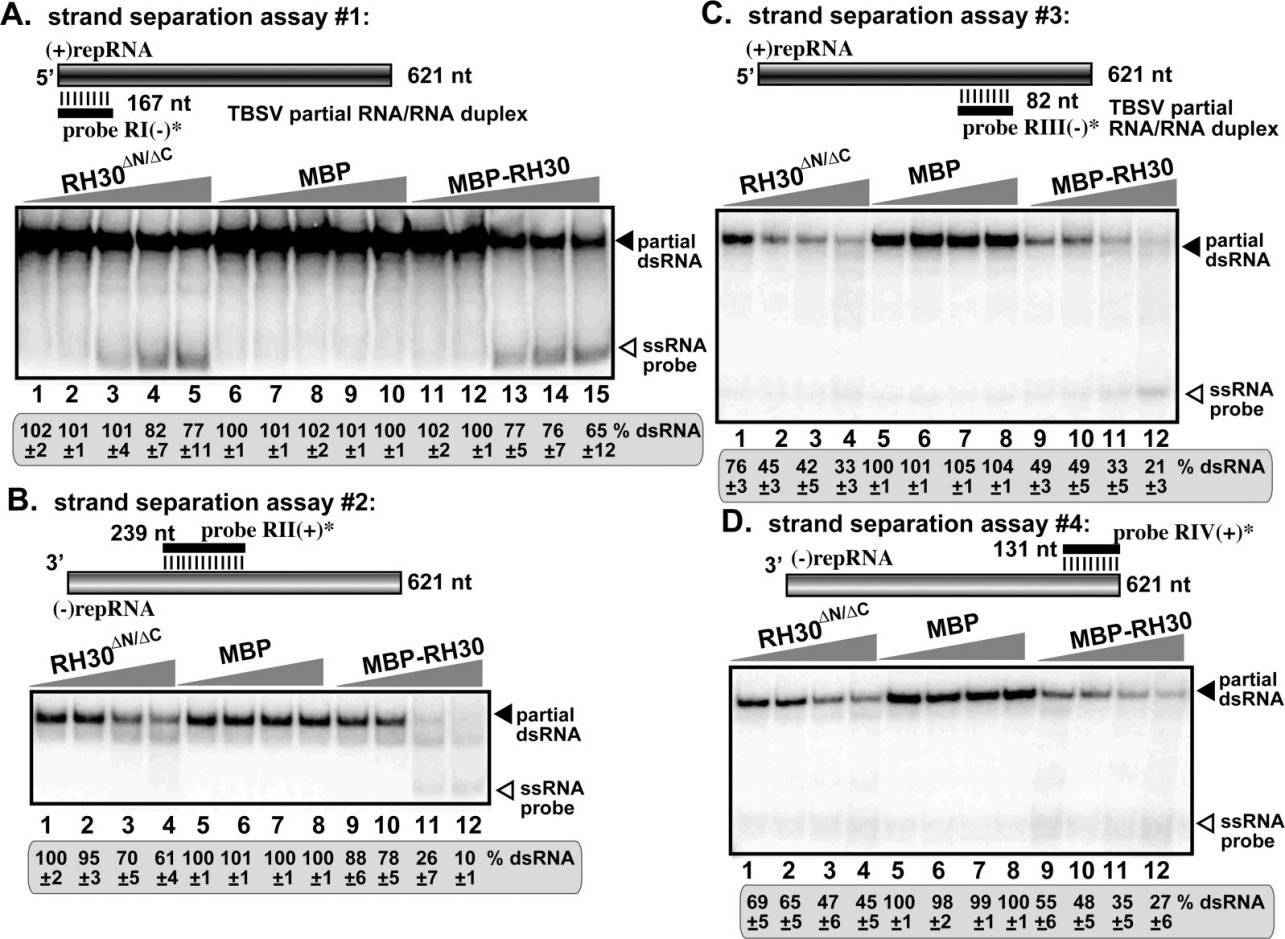
Decreased level of unwinding of the dsRNA region containing the RII(+)-SL *cis*-acting element involved in RNA template selection by RH30^ΔN/ΔC^ helicase in vitro. (A) Top: Schematic representation of the partial RNA/RNA duplexes used in the strand separation assay. The partial dsRNA template consists of unlabeled DI-72 (+)repRNA and a short ^32^P-labeled complementary (-)RNA (representing 169 nt RI of DI-72 repRNA), which anneals to the 621 nt DI-72 (+)repRNA. Increasing amounts (0.95, 1.9, 3.8, 7.6 and 11.4 μM) of purified recombinant MBP-RH30^ΔN/ΔC^, MBP-RH30, or MBP, as a control, were added to the reactions in the presence of ATP. Bottom: Representative native gel of ^32^P-labeled RNA products after the in vitro strand separation assay. Quantification of the partial dsRNA probe was done with a phosphorimager and Image QTL software. (B-D) Comparable strand separation assays using different regions, each representing a known *cis*-acting replication element, of the repRNA as shown. See panel A for further details. These experiments were repeated at least two times.

The antiviral activity of the full-length RH30 DEAD-box helicase is attributed to the efficient unwinding of the secondary structure of the RII(+) region in the TBSV (+)RNA [31]. RII(+) contains a critical cis-acting stem-loop element, termed RII(+)SL, which is required for the p33-mediated recruitment of the TBSV (+)RNA template into replication [16], and for the activation of the p92 RdRp [13]. Therefore, we tested if the purified RH30^ΔN/ΔC^ helicase could unwind partial dsRNA substrates in a dsRNA-strand separation assay [29,31], where parts of the TBSV repRNA were double-stranded as shown schematically in Fig 4. Interestingly, unlike the full-length RH30 helicase, the RH30^ΔN/ΔC^ helicase was found to inefficiently separate the partial dsRNA template, involving the RII sequence, in the presence of ATP (Fig 4B, lanes 1–4 versus 9–12). These findings suggest that, in contrast with the full-length RH30 helicase, the RH30^ΔN/ΔC^ helicase cannot change the RII(+)SL hairpin structure, thus it might not block the binding of TBSV p33 replication protein to the critical RII(+)-SL RNA recognition element required for template recruitment into replication. This could explain the lost antiviral activity of the RH30^ΔN/ΔC^ helicase.

However, RH30^ΔN/ΔC^ helicase was able to unwind the RI-containing partial dsRNA in vitro (Fig 4A, lanes 1–4 versus 5–8). Helicase-driven unwinding of the dsRNA structure within RI sequence of the dsRNA replication intermediate is critical during initiation of (+)-strand RNA synthesis [29,51]. The RI(-) region contains the promoter for (+)RNA synthesis, including loading of the p92^pol^ on the (-)RNA template [55]. We note that the unwinding activity of RH30^ΔN/ΔC^ helicase was comparable to that of the full-length RH30 helicase when tested with RIII and RIV (Fig 4C and 4D). Altogether, this ability of RH30^ΔN/ΔC^ helicase to unwind RI dsRNA structure, while inefficient on the RII dsRNA structure could explain its acquired pro-viral function by stimulating viral (+)-strand synthesis without inhibiting (+)RNA template selection/recruitment by p33 replication protein (see discussion).

**Fig 5:**
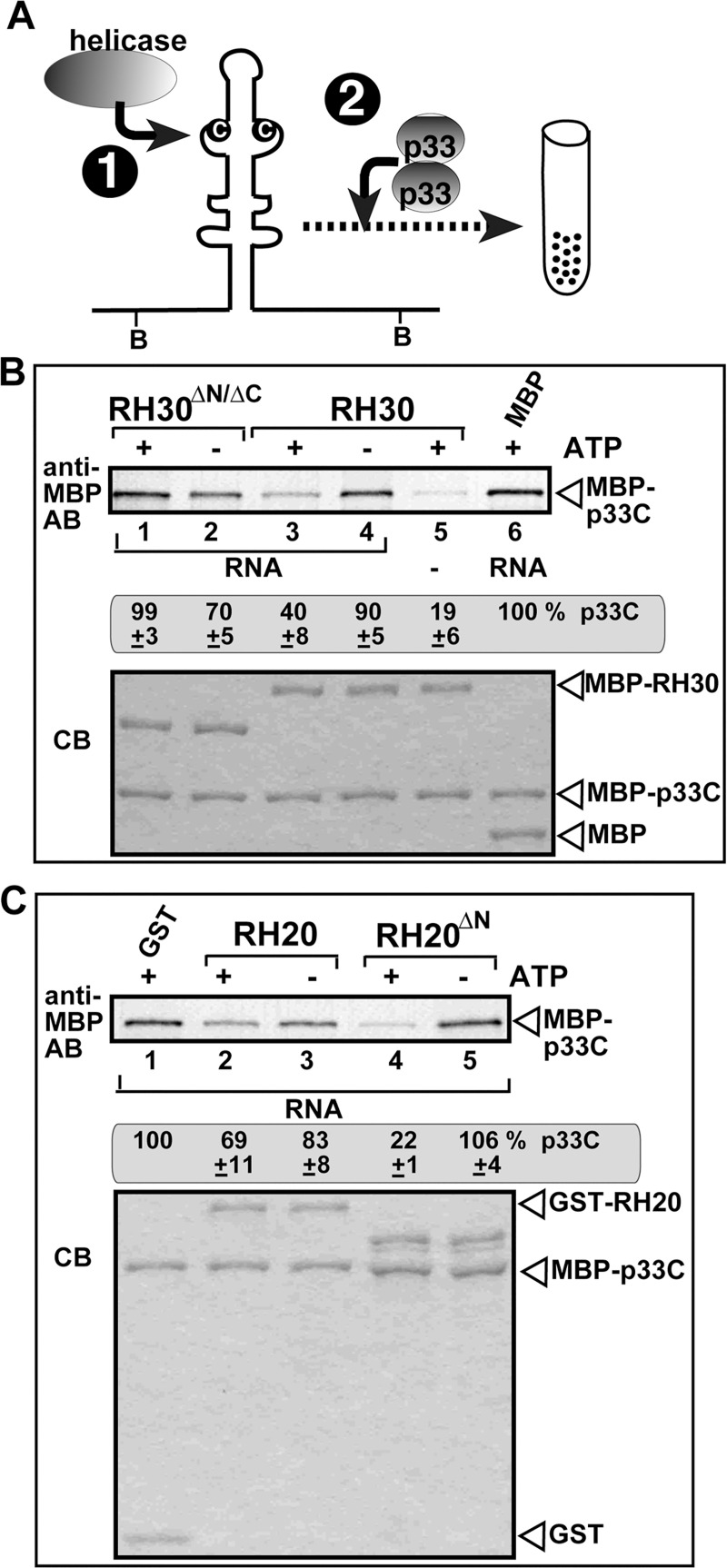
The effect of DEAD-box helicase mutants on the binding of p33 replication protein to a viral (+)RNA in vitro. (A) Top: Scheme of the in vitro p33-RNA capturing assay with biotinylated RII(+) RNA from TBSV bound to streptavidin-coated magnetic beads. The biotin-labeled RII(+) RNA probe (0.1 μg) and the purified helicase (3.8 μM) were allowed to form an RNP complex in the presence or absence of 1 mM ATP for 15 min, followed by addition of MBP-p33C protein (1.9 μM), and additional incubation for 15 min. Then, the biotin-labeled RII(+) RNA-protein complex was captured on streptavidin-coated magnetic beads, which were sequentially washed with a buffer. (B-C) The bound proteins were eluted from the beads, followed by measuring the amounts of MBP-p33C in the eluates by western blotting using anti-MBP antibody. Reduced amounts of MBP-p33C in the eluates mean that the given helicase inhibited the binding of p33C to the viral RNA, likely due to remodeling the RNA structure that could not be recognized by p33 replication protein. Nonbiotinylated RNA (panel B, lane 5) was used as a control. Bottom panels show SDS-PAGE analysis of the purified recombinant proteins stained by coomassie-blue. Each experiment was repeated three times.

To further characterize the lost antiviral function of RH30^ΔN/ΔC^ helicase during tombusvirus replication, we tested if RH30^ΔN/ΔC^ helicase could inhibit the selective binding of p33 replication protein to the viral RNA template *in vitro*. We used our recently developed p33-RNA capturing assay [31] based on a biotin-labeled RII(+) sequence of the TBSV (+)RNA, which represents RII(+)-SL RNA recognition element required for template recruitment into replication by the p33 replication protein [16]. RII(+)-SL of the TBSV RNA is also an essential assembly platform for the replicase complex [56]. Briefly, the biotin-labeled RII(+) RNA was pre-incubated either with purified RH30^ΔN/ΔC^ or RH30 helicases (Fig 5A). Then, we added the purified recombinant TBSV p33C (the soluble C-terminal region, including the RNA-binding and p33:p33/p92 interaction region of the p33 replication protein), which can bind specifically to RII(+)-SL carrying the C•C mismatch in the internal loop [16]. Subsequently, the biotin-labeled RII(+) RNA was captured on streptavidin-coated magnetic beads. The helicase and p33C proteins bound to the RII(+) RNA were eluted from the beads, followed by western blot analysis with anti-MBP antibody. This assay revealed that RH30^ΔN/ΔC^ did not inhibit the binding (thus capturing) of p33C to RII(+) RNA (Fig 5B, lanes 1–2 versus lane 6). This is in contrast with the antiviral full-length RH30 helicase, which strongly inhibited the binding of p33C to RII(+) RNA in the presence of ATP (Fig 5B, lane 3 versus lane 6) [31]. Based on these data, we suggest that RH30^ΔN/ΔC^ has lost its ability to interfere with the selective binding of p33 replication protein to the (+)RNA template. This lost ability of RH30^ΔN/ΔC^ is likely due to its inability to change RII(+) RNA structure. Indeed, this result is in agreement with the dsRNA unwinding data, which showed that RH30^ΔN/ΔC^ cannot efficiently separate the RII duplex (Fig 4B). Altogether, these observations could explain why RH30^ΔN/ΔC^ has no viral restriction function, while the full-length RH30 likely prevents the selective recognition/recruitment of TBSV (+)RNA by the p33 replication protein [31]. In addition, we propose that RH30^ΔN/ΔC^ acquired pro-viral function by selectively unwinding dsRNA structures within the RI segment required for TBSV (+)RNA initiation [28,57].

### The pro-viral role of the N-terminal domain of the cellular RH20 DEAD-box RNA helicase in tombusvirus replication

**Fig 6:**
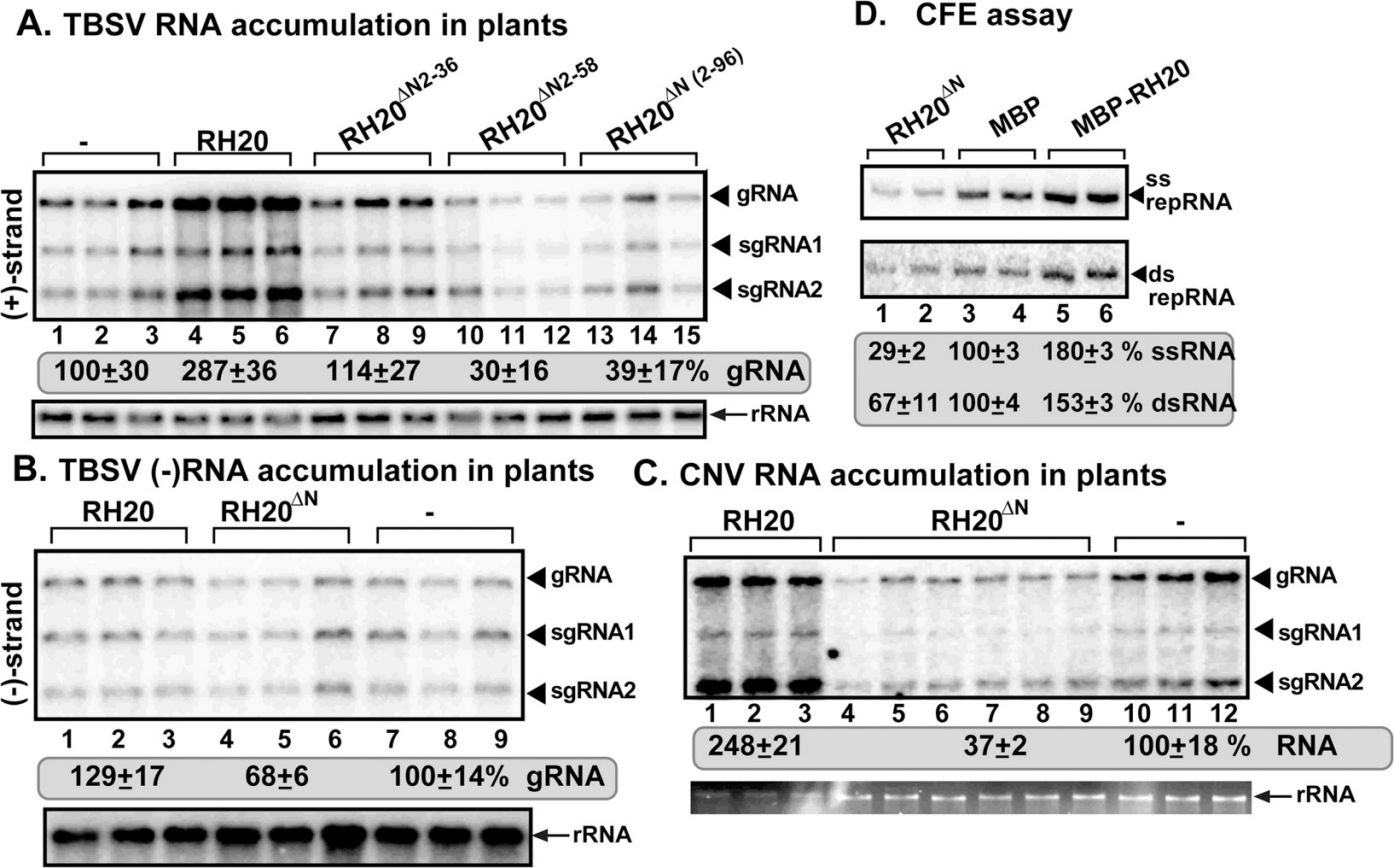
Effects of expression of truncation mutants of the pro-viral RH20 DEAD-box helicase on tombusvirus genomic (g)RNA replication in *N*. *benthamiana* plants. (A) *N*. *benthamiana* plants transiently expressing the N-terminal truncation derivatives of RH20 helicase were inoculated with TBSV. Top panel: Northern blot analyses of tombusvirus gRNA using a 3’ end specific probe shows the accumulation level of gRNA and subgenomic RNAs in plants expressing RH20 helicase and its derivatives when compared with control plants. Bottom panel: Northern blot shows 18S ribosomal RNA as a loading control. (B) Northern blot analyses of TBSV gRNA using a 3’ end specific probe for (-)RNA detection. TBSV (-)RNA levels were normalized to 18S rRNA levels. In comparison of full-length RH20 and the control samples, the statistical test shows P< .0001. In comparison of RH20^ΔN^ and the control samples, the statistical test shows P = .0485. (C) *N*. *benthamiana* plants expressing RH20^ΔN^ or RH20 helicases were inoculated with CNV. See further details in panel A. (D) An in vitro replicase reconstitution assay shows the inhibitory effect of RH20^ΔN^ helicase on TBSV repRNA accumulation. The purified recombinant tombusvirus p33 and p92^pol^ replication proteins from *E*. *coli* were added in combination with the template (+)repRNA to program the yeast-based CFE assay. The affinity-purified recombinant MBP-RH20^ΔN^ (1.9 μM), MBP-RH20 (1.9 μM each) or MBP (1.9 μM), as a control, were added to the reactions. Non-denaturing PAGE shows the accumulation of ^32^P-labeled (+)repRNAs and the dsRNA replication intermediate products made by the reconstituted replicases. Each experiment was repeated three times. In comparison of MBP-RH20^ΔN^ and MBP control samples, the statistical test shows P = .038. In comparison of MBP-RH20 and MBP control samples, the statistical test shows P = .0021.

To learn if the above findings of reversing the viral function of an *Arabidopsis* DEAD-box RNA helicase could be generalized, we also tested the pro-viral DDX3-like RH20 DEAD-box RNA helicase [29,51]. To identify what domains are responsible for the pro-viral function of RH20 DEAD-box RNA helicase, we expressed deletion mutants of RH20 in *N*. *benthamiana* plants via agroinfiltration. Expression of a short N-domain truncation mutant of RH20 led to elimination of the pro-viral activity of the RH20 helicase in TBSV replication in the inoculated leaves (RH20^ΔN2-36^, S2 Table). More importantly, expression of the entire N-terminal domain deletion mutant of RH20 helicase in *N*. *benthamiana* plants inhibited TBSV RNA accumulation by almost 3-fold (RH20^ΔN2-96^, S2 Table and Fig 6A, lanes 13–15 versus 1–3), indicating an antiviral activity. This suggests that RH20 requires the N-terminal domain to be an active pro-viral host factor in TBSV replication. Replication of other tombusviruses, such as CNV (Fig 6C) and CIRV (S2 Table) was also inhibited by 2-to-3-fold in *N*. *benthamiana* by the transient expression of the RH20^ΔN2-96^ missing the N-terminal domain (this mutant will be named RH20^ΔN^ below and in the figures). In contrast, deletion of the short C-terminal domain in RH20 did not interfere with the pro-viral function in *N*. *benthamiana* (RH20^ΔC^, S2 Table). Overall, in the absence of its N-terminal domain, the pro-viral RH20 DEAD-box helicase gains a new antiviral function. Thus, similar to the case with RH30 helicase, the functional identity of pro-viral RH20 helicase could be reversed by removing a regulatory domain.

### The antiviral RH20^ΔN^ DEAD-box helicase inhibits tombusvirus RNA synthesis *in vitro*

To learn what features of RH20^ΔN^ helicase caused it to become antiviral, we affinity-purified the recombinant RH20^ΔN^ helicase from *E*. *coli*, and tested its activity *in vitro* in a TBSV replicase reconstitution assay [18,54]. Addition of the purified RH20^ΔN^ helicase to the replicase reconstitution assay resulted in decreased TBSV repRNA replication by ~3-fold (Fig 6D, lanes 1–2 versus lanes 5–6). The double-stranded repRNA replication intermediate was also reduced by ~40% in the presence of RH20^ΔN^ helicase in comparison with the full-length RH20 helicase. Based on these data, we suggest that RH20^ΔN^ helicase likely inhibits both (-) and (+)-strand RNA synthesis during TBSV replication. This is in agreement with *in planta* data on TBSV accumulation, which also showed decreased levels of both (-)RNAs and (+)RNAs when RH20^ΔN^ helicase was expressed in *N*. *benthamiana* (Fig 6A, lanes 13–15 and Fig 6B, lanes 4–6).

### The antiviral RH20^ΔN^ DEAD-box helicase is re-targeted into the tombusvirus replication compartment in plants

**Fig 7:**
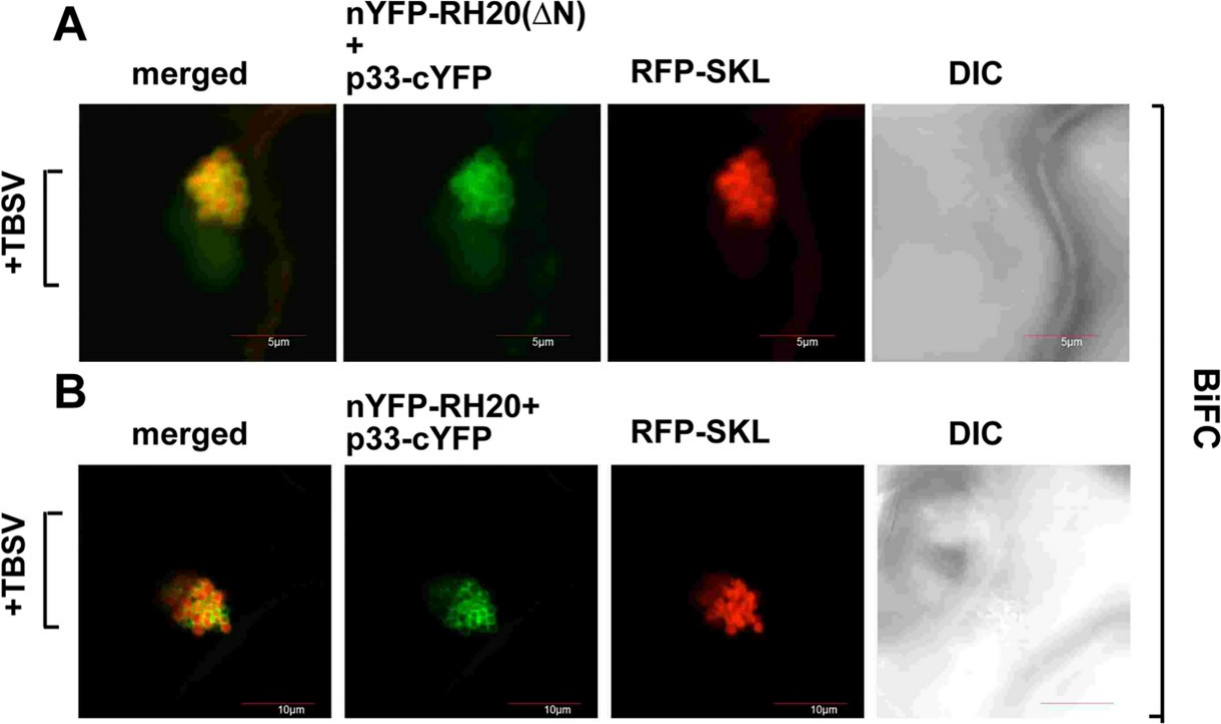
Interaction between RH20^ΔN^ helicase and the TBSV replication protein in plants. (A-B) Interactions between TBSV p33 replication protein and the RH20^ΔN^ helicase (panel A) or the full-length RH20 (panel B) were detected by BiFC. The TBSV p33-cYFP replication protein and the nYFP-RH20^ΔN^, nYFP-RH20 and the RFP-SKL peroxisomal marker protein were expressed via agroinfiltration in *N*. *benthamiana*. The merged image shows the efficient co-localization of the peroxisomal RFP-SKL with the BiFC signals, indicating that the interactions between the tombusvirus p33 replication protein and the recruited RH20^ΔN^ and RH20 helicases occur in the large VROs, which consist of aggregated peroxisomes. Scale bars represent 10 μm.

To test if the antiviral RH20^ΔN^ helicase is present in the replication compartment, we conducted BiFC experiments in *N*. *benthamiana* leaves. The antiviral RH20^ΔN^ helicase, similar to the pro-viral full-length RH20 helicase, also showed interaction with the TBSV p33 replication protein within the viral replication compartment, marked by the peroxisomal matrix marker RFP-SKL (Fig 7A and 7B). Thus, both the pro-viral full-length RH20 and antiviral truncated RH20^ΔN^ helicases are re-targeted to the VROs during TBSV replication.

### The antiviral RH20^ΔN^ DEAD-box helicase efficiently unwinds critical cis-acting elements in the TBSV RNA

**Fig 8:**
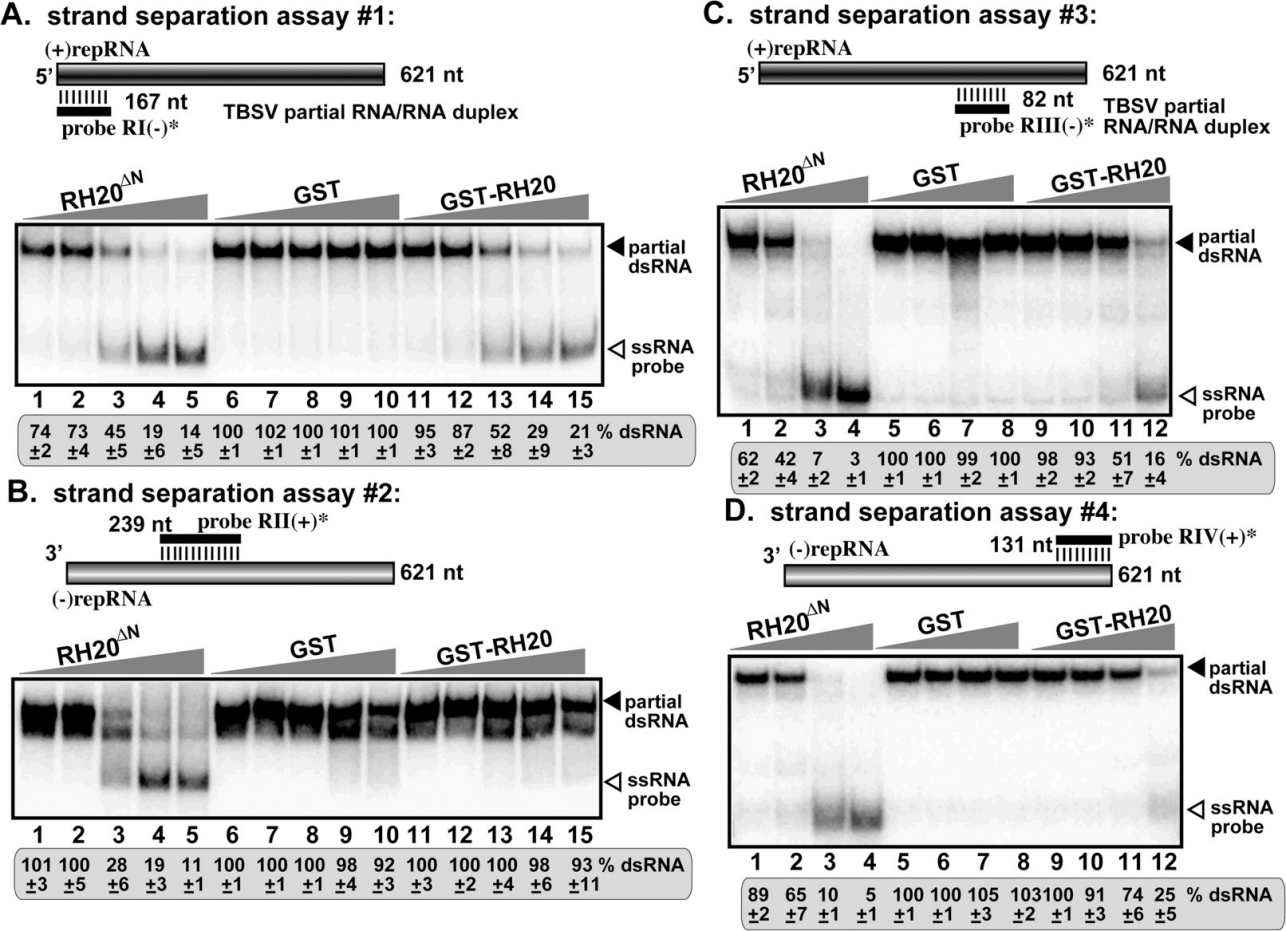
Enhanced level of unwinding of the dsRNA region containing the RII(+)-SL *cis*-acting element involved in RNA template selection by RH20^ΔN^ helicase in vitro. (A-D) On the top for each panel, we show schematically the annealed partial RNA/RNA duplexes used in the strand separation assay. Increasing amounts of purified recombinant GST-RH20^ΔN^ helicase, GST-RH20, or GST, as a control, were added to the reactions in the presence of ATP. See Fig 4 for further details.

Here, we again used the *in vitro* strand-separation assay to test the antiviral activity of the RH20^ΔN^ helicase. Surprisingly, we observed a dramatically enhanced unwinding activity of RH20^ΔN^ helicase in comparison with the pro-viral full-length RH20 helicase. For, example, the purified RH20^ΔN^ helicase, unlike the full-length RH20 helicase, could efficiently unwind the partial dsRNA template, involving the critical RII sequence, in the presence of ATP (Fig 8B, lanes 1–5 versus 11–15). These findings suggest that, in contrast with the full-length RH20 helicase, the RH20^ΔN^ helicase can ‘destroy” the RII(+)SL hairpin structure, thus it might be able to block the binding of TBSV p33 replication protein to the critical RII(+)-SL RNA recognition element, which event is required for template recruitment into replication. Actually, RH20^ΔN^ helicase was able to efficiently unwind all the TBSV dsRNA templates provided (Fig 8). This efficient unwinding of viral RNA structures by RH20^ΔN^ helicase likely is the reason for its antiviral function. The pro-viral full-length RH20 is more selectively targets RI sequence, which is critical during initiation of (+)-strand RNA synthesis, but not that of RII of the dsRNA replication intermediate [29,51]. Therefore, we suggest that the RH20^ΔN^ helicase becomes antiviral based on its lost RNA sequence selectivity in comparison with the more selective pro-viral full-length RH20 helicase.

To further characterize the acquired antiviral function of RH20^ΔN^ helicase during tombusvirus replication, we again used our p33-RNA capturing assay [31] based on a biotin-labeled RII(+) sequence. The biotin-labeled RII(+) RNA was pre-incubated either with purified RH20^ΔN^ or RH20 and subsequently, with the purified TBSV p33C as described above (Fig 5A). This assay revealed that RH20^ΔN^ strongly inhibited the binding of p33C to the RII(+) RNA in the presence of ATP (Fig 5C, lane 4 versus lane 1). On the contrary, the full-length RH20 did not inhibit efficiently the binding of p33C to the RII(+) RNA (Fig 5C, lanes 2–3 versus lane 1). Based on these data, we suggest that RH20^ΔN^ acquired a new ability to interfere with the selective binding of p33 replication protein to the (+)RNA template. This observation could explain why RH20^ΔN^ obtained a new viral restriction function, while the full-length RH20 does not likely prevent the selective recognition/recruitment of TBSV (+)RNA by the p33 replication protein [31]. These data also invite the attention to the possible roles of cellular helicases to unwind the hairpin structure of a *cis*-replication element in TBSV (+)RNA to prevent its selective recognition and recruitment into VRC by the p33 replication protein, which is an early step in replication (see discussion).

### Chimeric DEAD-box helicases confirm the critical roles of the N-terminal domains in DEAD-box helicases in TBSV replication

**Fig 9:**
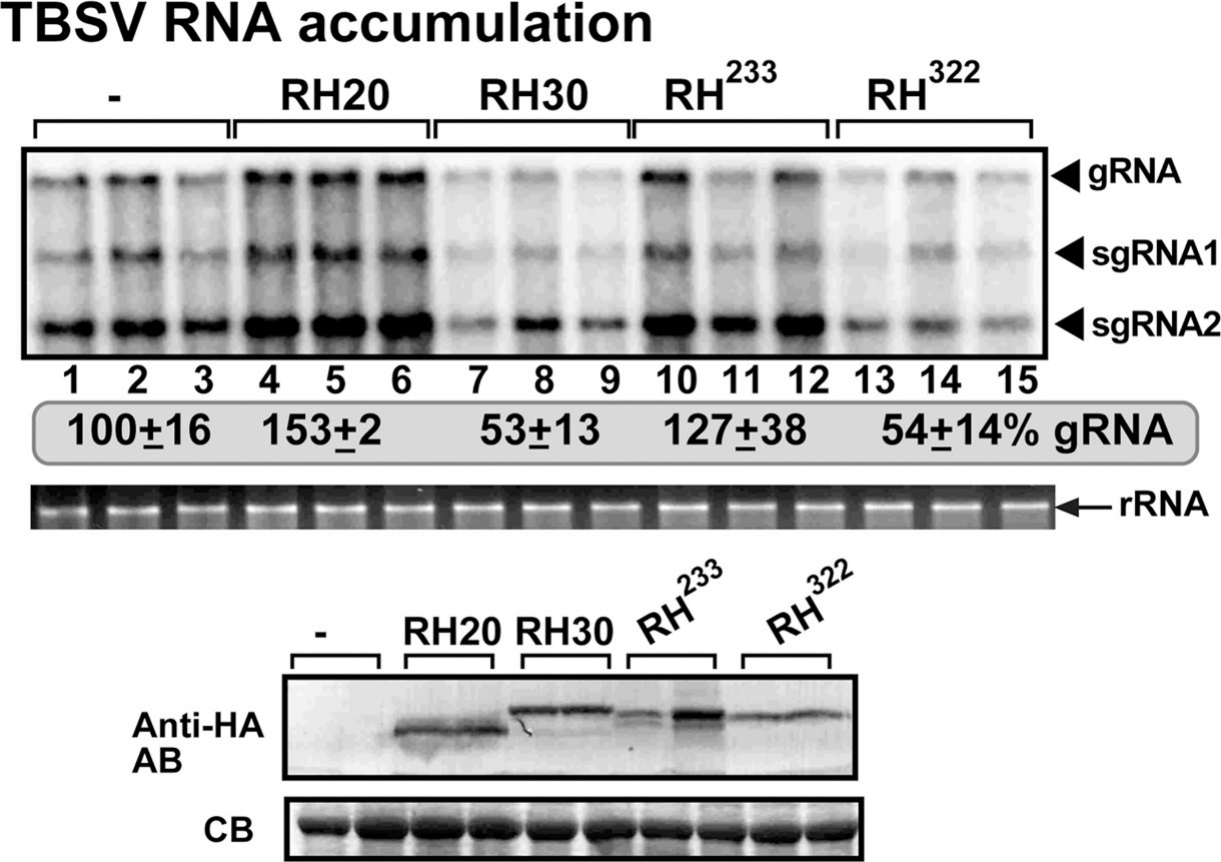
Effects of expression of chimeric DEAD-box helicases on TBSV replication in *N*. *benthamiana* plants. Top panel: Schematic representation of the chimeric helicases constructed based on RH20 and RH30 helicases. The chimeric helicases either contained the N-terminal domain of RH20 combined with the core helicase domain and C-terminal domain of RH30 (namely RH^233^) or the N-terminal domain of RH30 combined with the core helicase domain and C-terminal domain of RH20 (namely RH^322^). The plant leaves transiently expressing the chimeric derivatives of RH20 and RH30 helicases were inoculated with TBSV. Top panel: Northern blot analysis of tombusvirus gRNA using a 3’ end specific probe shows the accumulation level of gRNA and subgenomic RNAs at 1.5 days post-inoculation. Bottom panels: Western blot analysis of the level of HA-tagged chimeric derivatives of RH20 and RH30 helicases with anti-HA antibody. Each experiment was repeated at least three times.

To obtain further evidence on the critical roles of the N-terminal domains of RH20 and RH30 DEAD-box helicases in specifying their functions in TBSV replication, we constructed two chimeric DEAD-box helicases by switching the N-terminal domains between RH20 and RH30 (Fig 9). The chimeric helicase containing the N-terminal domain from RH30 and the remaining core helicase and C-terminal sequences from RH20 (i.e., named as RH^322^ helicase) showed antiviral activity against TBSV when expressed in *N*. *benthamiana* (Fig 9, lanes 13–15 versus 1–3). Thus, the chimeric RH^322^ has anti-TBSV activity similar to the full-length RH30 helicase (Fig 9, lanes 13–15 versus 7–9). On the contrary, the other chimeric helicase containing the N-terminal domain from RH20 and the remaining sequences from RH30 (i.e., named as RH^233^ helicase) showed pro-viral activity on TBSV replication when expressed in *N*. *benthamiana* (Fig 9, lanes 10–12 versus 1–3). These results support the notion that the N-terminal domain in the DEAD-box helicases might be an important determinant of the specific pro-viral or antiviral roles of these DEAD-box helicases in TBSV replication.

### The N-terminal-domain in the antiviral RH30 DEAD-box helicase is required to interfere with the interaction between RH20 helicase and the p33 replication protein

**Fig 10:**
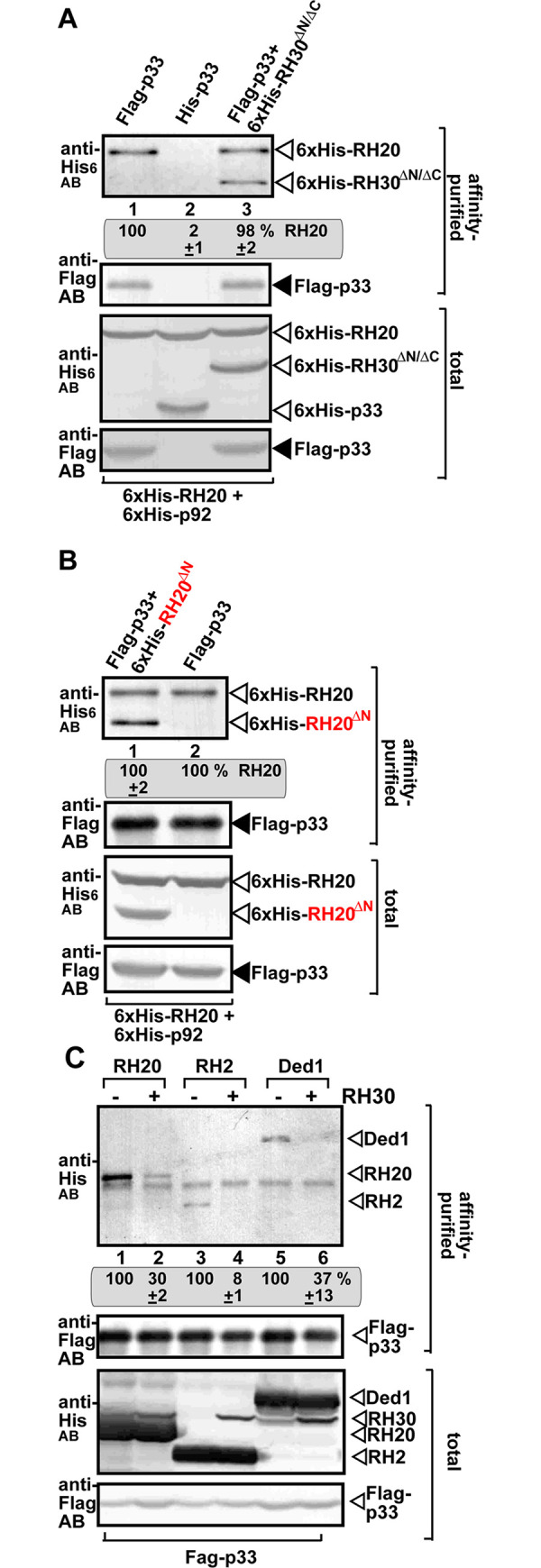
The truncated helicases do not inhibit the interaction between the pro-viral RH20 helicase and the p33 replication protein. (A) Co-purification of His_6_-tagged RH20 and RH30^ΔN/ΔC^ with Flag-p33 replication protein from subcellular membranes of yeasts. Top panels: Western blot analysis of co-purified His_6_-RH20 and His_6_-RH30^ΔN/ΔC^ (lane 3) with Flag-affinity purified Flag-p33 replication protein. Expression of His_6_-p33 was used as a control (lane 2). His_6_-RH30^ΔN/ΔC^ and His_6_-RH20 were detected with anti-His antibody, while Flag-p33 replication protein was detected with anti-FLAG antibody. Bottom panels: blot of total His_6_-p33, His_6_-RH30^ΔN/ΔC^ and His_6_-RH20 and Flag-p33 in the total yeast extracts detected with anti-His and anti-Flag antibodies, respectively. (B) Co-purification of His_6_-tagged RH20 and His_6_-RH20^ΔN^ with Flag-p33 replication protein from subcellular membranes of yeasts. See further details in panel A. (C) Co-purification of His_6_-tagged RH20, His_6_-RH2 and His_6_-Ded1 with Flag-p33 replication protein from subcellular membranes of yeasts. Yeasts were co-expressing His_6_-RH30 in samples shown. See further details in panel A.

An important feature of both antiviral RH30 and the pro-viral RH20 helicases is to bind to the TBSV p33 replication protein [29,31,51]. Therefore, it is possible that the antiviral DEAD-box helicases are inhibitory to TBSV replication due to their predicted abilities to out compete the pro-viral RH20 helicase for the interaction with the p33 replication protein. This model was tested in co-purification experiments from yeast. Co-expression of the truncated antiviral RH20^ΔN^ helicase with the pro-viral full-length RH20 resulted in similar amounts of co-purified RH20 with the p33 replication protein from yeast membranes (Fig 10A, lanes 3 versus 1). Co-expression of the truncated pro-viral RH30^ΔN/ΔC^ helicase with the pro-viral full-length RH20 also resulted in similar amounts of co-purified RH20 with the p33 replication protein from yeast membranes (Fig 10B). Thus, the co-expression of these truncated DEAD-box helicases did not change the amount of co-purified RH20 with the p33 replication protein from yeast membranes. Therefore, it is unlikely that the acquired antiviral function of RH20^ΔN^ helicase or the pro-viral RH30^ΔN/ΔC^ helicase is based on altering the interaction between the pro-viral RH20 helicase and the TBSV p33 replication protein.

On the contrary, we found that co-expression of the full-length antiviral RH30 helicase with the pro-viral RH20 resulted in reduced amounts of co-purified RH20 with the p33 replication protein from yeast membranes (Fig 10C, lanes 2 versus 1). Similarly, co-expression of the full-length antiviral RH30 with the pro-viral eIF4AIII-like RH2 helicase or the yeast DDX3-like Ded1 helicase [an ortholog of the plant RH20 DEAD-box helicase with similar pro-viral function [29,51] resulted in reduced amounts of co-purified RH2 or Ded1 helicases with the p33 replication protein from yeast membranes (Fig 10C). Thus, this is in contrast with the results obtained when we co-expressed the truncated antiviral RH20^ΔN^ with the pro-viral RH20 in yeasts. Based on these results, it seems that the truncated DEAD-box helicases lack the ability to compete with the pro-viral RH20 helicase to interact with the p33 replication protein, whereas the full-length antiviral RH30 helicase has this ability. This could explain why the full-length RH30 has a much more potent inhibitory activity than RH20^ΔN^ on TBSV replication in *N*. *benthamiana* (see below).

### Dominant effects of the antiviral DEAD-box helicases over the pro-viral RH20 DEAD-box helicase in TBSV replication

**Fig 11:**
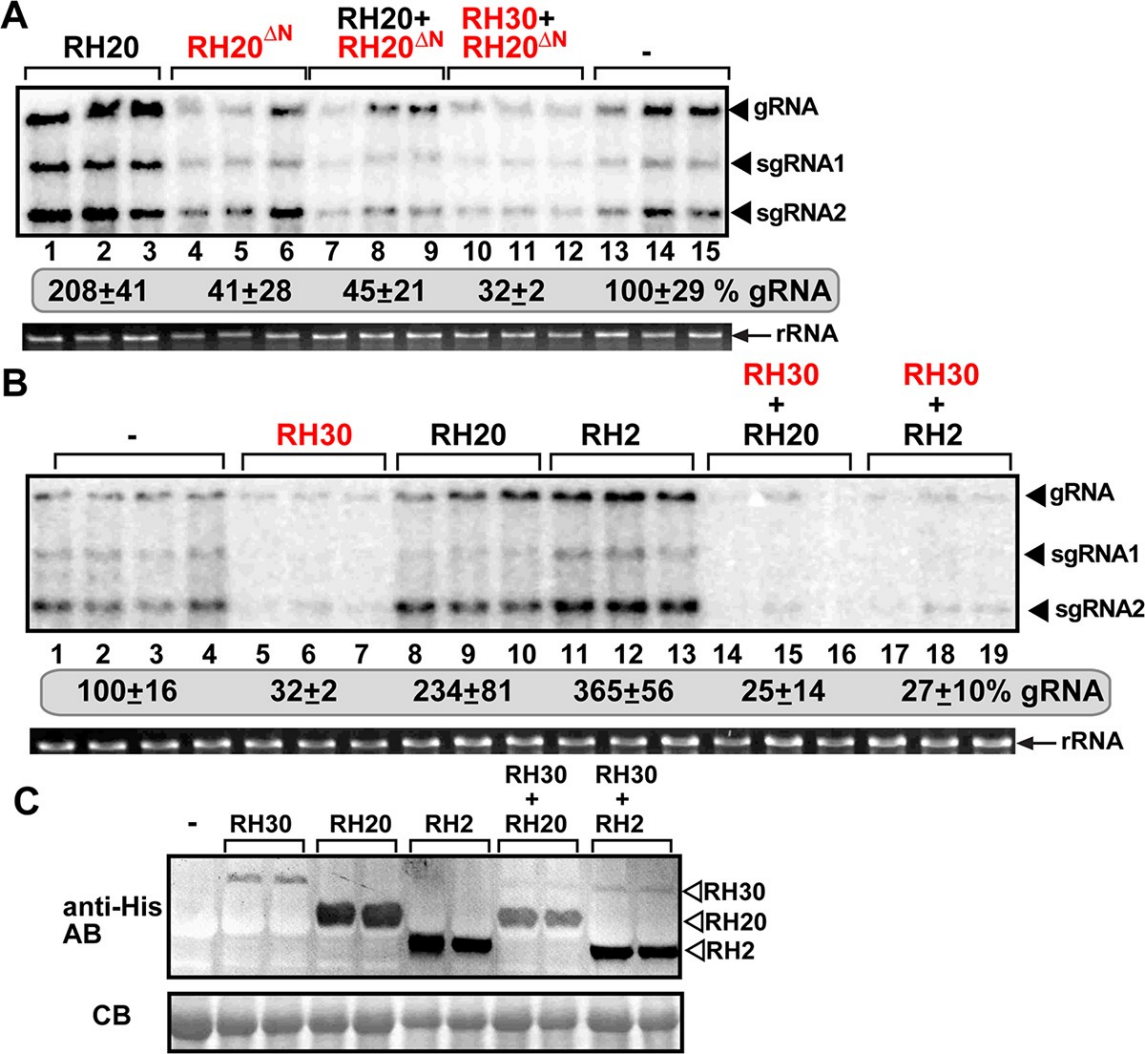
Effects of co-expression of antiviral helicases and pro-viral helicases on TBSV replication in *N*. *benthamiana* plants. (A) The plant leaves, which were inoculated with TBSV, transiently co-expressed the pro-viral RH20 and the novel antiviral RH20^ΔN^ helicases, as shown. Top panel: Northern blot analysis of tombusvirus gRNA using a 3’ end specific probe shows the accumulation level of gRNA and subgenomic RNAs. (B) The plant leaves, which were inoculated with TBSV, transiently co-expressed the pro-viral His_6_-RH20, His_6_-RH2 and antiviral His_6_-RH30 helicases, as shown. See further details in panel A. (C) Western blot analysis shows the accumulation of the expressed helicases using anti-His antibody. Each experiment was repeated at least three times.

Through reversing the pro-viral role of RH20 DEAD-box helicase into an antiviral role in the RH20^ΔN^ helicase, we created a novel antiviral tool. To test how effective is this new antiviral tool, we asked the interesting question whether RH20^ΔN^ helicase could compete with the pro-viral RH20 to affect TBSV replication. Interestingly, co-expression of RH20 with RH20^ΔN^ helicase resulted in reduced TBSV RNA accumulation in *N*. *benthamiana* (Fig 11A, lanes 7–9 versus 1–3). Because these data suggested that the antiviral function of a DEAD-box helicase could be a dominant feature over the pro-viral RH20 helicase, we also co-expressed the full-length antiviral RH30 with the pro-viral RH20 in *N*. *benthamiana* infected with TBSV. We found that TBSV replication was reduced to a similar level as it was observed with the expression of RH30 helicase alone (Fig 11B, lanes 14–16 versus 5–7 and 8–10). Co-expression of the full-length antiviral RH30 with another pro-viral DEAD-box helicase, namely eIF4AIII-like RH2 helicase, which function differently from RH20 by enhancing the activity of a replication enhancer element in the viral (-)RNA [28], also resulted in TBSV replication at a highly reduced level (Fig 11B. lanes 17–19). Thus, it seems that the antiviral DEAD-box helicases, both the full-length RH30 and truncated version (RH20^ΔN^ helicase), acted in a dominant manner over the pro-viral RH20 and RH2 DEAD-box helicases in plants. The dominant effect over the pro-viral RH20 DEAD-box helicase by the antiviral RH30 and RH20^ΔN^ in TBSV replication is likely due to their abilities to unwind the critical RII(+)-SL *cis*-acting RNA element, which is an early step in replication (see discussion). Nevertheless, the dominant nature of antiviral helicases over pro-viral RNA helicases shows a great potential to develop new antiviral factors. This aspect of cellular DEAD-box helicases will need to be further studied after the functions of more cellular DEAD-box helicases in TBSV replication are characterized in the future.

## Discussion

Unlike many other pathogens, (+)RNA viruses code for only a rather limited number of genes, making them highly dependent on numerous co-opted host factors for supporting viral replication and other viral processes during their infections. This extreme dependence on subverted host factors, however, renders (+)RNA viruses vulnerable to host restriction factors that could block virus replication [58]. Accordingly, tombusviruses are dependent on the cellular DDX3-like RH20 and eIF4AIII-like RH2 DEAD-box helicases during their replication in plants [27–29,51]. However, there are over a hundred RNA helicases coded in plant genomes [36,59], which might not assist tombusvirus replication, but instead, might block the viral replication process. Indeed, we have previously shown that the DDX17-like RH30 helicase, which is rather similar to the pro-viral DDX3-like RH20 helicase, has strong anti-tombusvirus activity in plants [31]. However, it is currently incompletely defined what features make particular DEAD-box helicases either pro-viral or antiviral.

In this work, we have unraveled that the functional identity/orientation of the co-opted DEAD-box helicases could be altered by changing the pro-viral RH20 helicase into antiviral and the antiviral RH30 helicase into a pro-viral helicase via deletion of regulatory sequences of these cellular helicases or making helicase chimeras. Notably, we find that the unique N-terminal domains in RH20 and RH30 DEAD-box helicases seem to be really important to specify the activity of the given cellular helicase. In the absence of the N-terminal domain, the core helicase domain becomes unhinged, showing altered specificity in RNA duplex unwinding activities. We note that the RNA helicase activity, however, seems to be important for both pro-viral and antiviral activities, because deletion or active site mutation of the core helicase domain eliminated these activities (S1 and S2 Tables). We also observed that the changed functional identity of the cellular helicases manifested with both the peroxisomal-replicating TBSV and the mitochondrial-replicating CIRV in plants.

**Fig 12:**
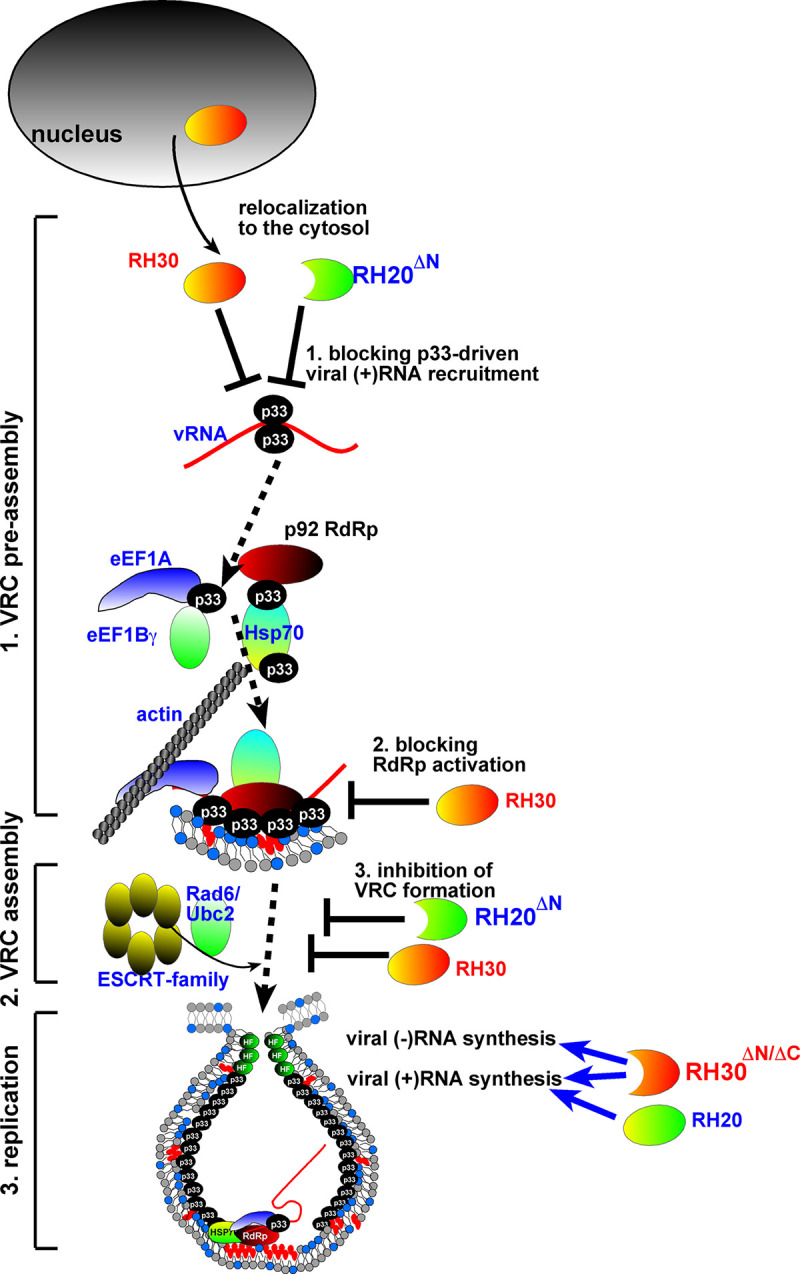
A model showing the acquired functions of the truncated helicases in TBSV replication. Based on our current and previous data, we propose that the RH20^ΔN^ helicase becomes a novel antiviral restriction factor by inhibiting the recruitment of the viral (+)RNA and, thus, blocking VRC formation. This inhibitory effect of RH20^ΔN^ helicase is likely through remodeling the structure of the RII(+)-SL *cis*-acting RNA element, which might block the specific recognition by p33 replication protein, which requires the RII(+)-SL stem-loop structure. RH20^ΔN^ helicase inhibits VRC assembly as well, because the stem-loop structure in RII(+)-SL is essential part of the VRC assembly platform. In contrast, RH30^ΔN/ΔC^ helicase becomes a novel pro-viral factor by opening up the RI-containing dsRNA structure within the dsRNA replication intermediate. This, in turn, RH30^ΔN/ΔC^ helicase likely facilitates the initiation of (+)-strand synthesis by the TBSV RdRp. We propose that unlike the full-length RH30, the RH30^ΔN/ΔC^ helicase has lost its ability to remodel RII(+)-SL structure, which is required for antiviral activity of RH30.

The tombusvirus genome is loaded with *cis*-acting RNA replication and translation elements to facilitate well-orchestrated and efficient viral replication [19]. The emerging picture from recent studies with TBSV is that cellular helicases could target various *cis*-acting elements in TBSV RNAs. Accordingly, in vitro studies revealed that one of the most important features of the cellular helicases is their ability to unwind the RNA structures within particular regions of the TBSV RNA. We find that the critical feature for the pro-viral DEAD-box helicases, such as RH30^ΔN/ΔC^ helicase (this work) and the full-length RH20, is to selectively target and unwind the RI sequence (5’UTR) of the dsRNA replication intermediate, which is critical during initiation of (+)-strand RNA synthesis [27–29,51]. On the contrary, the critical feature of the antiviral DEAD-box helicases, such as RH20^ΔN^ helicase (this work) and the full-length RH30 [31], is to efficiently target and “destroy” the RII(+)SL hairpin structure, and thus block the selective binding of TBSV p33 replication protein to the critical RII(+)-SL RNA recognition element, which is absolutely required for RNA template recruitment into replication, VRC assembly and viral RdRp activation (Fig 12) [13,14,16,60]. Indeed, we demonstrated that the antiviral RH20^ΔN^ (this work) and RH30 helicases [31] interfere with the binding of the p33 replication protein to the viral RII(+)RNA template in vitro, whereas the pro-viral RH30^ΔN/ΔC^ and RH20 helicases did not inhibit this process efficiently (Fig 5).

One of our interesting findings in this work is the observation of the dominant nature of the antiviral helicases over the pro-viral RH20 helicase. For example, although RH20^ΔN^ helicase facilitates the unwinding of RI-containing sequences, which are important for initiation of (+)RNA synthesis by the TBSV RdRp [29,51], this “pro-viral” feature of RH20^ΔN^ helicase might not be important in vivo. This is because the antiviral activity of RH20^ΔN^ helicase, involving unwinding of RII(+), which remodels RII(+)-SL and inhibits the recruitment of the viral RNA into replication, is an early step during TBSV replication (Fig 12). Thus, by unwinding the RII(+) RNA secondary structure and thus, inhibiting viral (+)RNA recognition by the p33 replication protein, both RH20^ΔN^ and the full-length RH30 helicases block this early step in tombusvirus replication. This makes this early event a dominant feature via preventing the predicted pro-viral potential of RH20^ΔN^ helicase or the pro-viral RH20 helicase (when RH30 is co-expressed) to facilitate the initiation of (+)RNA synthesis, which is a late step and depends on the (+)RNA requirement during the early steps.

The truncated cellular helicases with altered functional identity were different from the full-length helicases in biochemical features. For example, in contrast with the full-length RH30 helicase, the truncated RH30^ΔN/ΔC^ or RH20^ΔN^ helicases were not found to affect the important interaction between the pro-viral RH20 helicase and the p33 replication protein. Taken together, we propose that the major difference is that the antiviral and pro-viral helicases target different critical *cis*-acting regions within the viral RNA as discussed above.

Overall, the evolving features of the unique N-terminal domains of cellular DEAD-box helicases seem to be key components of the arms race between tombusviruses and their hosts. We propose that deletions/mutations in critical positions within the N-terminal domains of cellular DEAD-box helicases might render them either antiviral or pro-viral. The modular nature of the regulatory/specificity domains in cellular DEAD-box helicases might allow plants to gain new antiviral functions during evolution. Moreover, these findings will open up the possibility for science to turn pro-viral host factors into antiviral factors, for the benefit of agriculture and health science. We envision that the modular structure of regulatory/specificity domains in RNA helicases make them highly suitable to create numerous restriction factors from known pro-viral factors by altering existing regulatory sequences in these proteins. Alternatively, by using synthetic biology tools, future research could increase our arsenal of antiviral factors.

It will be interesting to learn if functions of helicases could be reversed in case of other RNA viruses and retroviruses. Those viruses also usurp several cellular DEAD-box helicases to facilitate their replication and other viral processes during infection [49,61]. Moreover, the host also deploys DEAD-box helicases to inhibit the replication of the above viruses [41,61]. Many of the identified DEAD-box helicases with restriction functions are conserved in plants and mammals.

**In summary:** We have demonstrated that the viral functions of the plant DDX17-like RH30 DEAD-box helicase and DDX3-like RH20 DEAD-box helicase could be reversed in tombusvirus replication by deletion of unique N-terminal domain or by making helicase chimeras. The discovery of the sequence plasticity within regulatory domains of DEAD-box helicases, which can alter recognition of different cis-acting elements in the viral genome, illustrates the evolutionary potential of RNA helicases in the arms race between viruses and their hosts, including key roles of RNA helicases in plant innate immunity.

## Materials and methods

Please find several basic approaches described in the supplementary S1 Text.

### The accumulation of viral RNAs in yeast and plants

To launch TBSV repRNA replication (based on DI-72 RNA) in yeast (*S*. *cerevisiae*) BY4741 wild-type (wt) strain, yeast cells were transformed with LpGAD-CUP1::HisFlag-p92 and HpGBK-CUP1::HisFlag-p33/GAL1::DI-72. The plasmids for expression of RH30 and its mutants, including pYES-vector (as a control), pYES-AtRH30, pYES-AtRH30^F416L^, pYES-AtRH30^ΔN^ or pYES-AtRH30^ΔHel/ΔC^, were also introduced into yeast cells, respectively. The obtained yeast transformants were grown in SC-ULH^-^ media supplemented with 2% galactose and 0.1 mM BCS at 23°C. After 18 h culturing, the yeast cultures were transferred to SC-ULH^-^ media supplemented with 2% galactose and 50 μM CuSO_4_ and incubated at 29°C. After 7 h, the obtained yeast cells were used for RNA and protein extractions, followed by northern blot analysis and western blot analysis as previously described [31].

To detect the accumulation of tombusviruses in *N*. *benthamiana* plants expressing *Arabidopsis* RH20, RH30 and their mutants, the leaves of *N*. *benthamiana* were infiltrated with agrobacterium cultures carrying pGD-P19 (OD_600_ 0.2) and pGD vector (OD_600_ 0.2, as a control for RH30 and its mutant), pGD-AtRH30 (OD_600_ 0.6), pGD-AtRH30^ΔN/ΔC^ (OD_600_ 0.6), pGD-3HA-AtRH20^ΔN2-36^ (OD_600_ 0.6), pGD-3HA-AtRH20^ΔN2-58^ (OD_600_ 0.6) or pGD-3HA-AtRH20^ΔN2-96^ (OD_600_ 0.6). In the experiments based on CNV infections, *N*. *benthamiana* plants were also co-infiltrated with agrobacterium carrying pGD-CNV^20Kstop^ (OD_600_ 0.2). For TBSV or CIRV infections, *N*. *benthamiana* plants were inoculated with TBSV or CIRV crude sap inocula at 16 h after agroinfiltration. Total RNA extraction and northern blot analysis were performed as described [28] to measure the accumulation levels of tombusviruses in inoculated leaves at 2.5 d post virus inoculation (dpi) for CNV or CIRV infections and at 1.5 dpi for TBSV infections. CNV and CIRV RNAs were detected by ^32^P-labled probes targeting the 3’-end of CNV (+)RNA and CIRV (+)RNA, respectively. The positive-sense and negative sense of TBSV RNAs were detected by specific ^32^P-labeled probes, respectively, targeting the 3’-ends of TBSV (+) and (-)RNAs, respectively. Western blot analysis was performed with anti-HA antibody.

To investigate the effects of co-expression of pro-viral and anti-viral RNA helicases on TBSV infections, the leaves of *N*. *benthamiana* were infiltrated with agrobacteria carrying pGD-P19 and different combinations of protein expression vectors as follows: (i) pGD-35S vector as a control (OD_600_ 0.8); (ii) pGD-RH30 (OD_600_ 0.4) and pGD-35S vector (OD_600_ 0.4); (iii) pGD-RH30ΔN/ΔC (OD_600_ 0.4) and pGD-35S vector (OD_600_ 0.4); (iv) pGD-RH30 (OD_600_ 0.4) and pGD-RH30ΔN/ΔC (OD_600_ 0.4); (v) pGD-RH30ΔN/ΔC (OD_600_ 0.4) and pGD-3HA-RH20 (OD_600_ 0.4); (vi) pGD-RH20 (OD_600_ 0.4) and pGD-35S vector (OD_600_ 0.4); (vii) pGD-RH20ΔN2-96 (OD_600_ 0.4) and pGD-35S vector (OD_600_ 0.4); (viii) pGD-RH20 (OD_600_ 0.4) and pGD-RH20ΔN2-96 (OD_600_ 0.4); (ix) pGD-RH20ΔN2-96 (OD_600_ 0.4) and pGD-RH30 (OD_600_ 0.4). Approximately 16 h post infiltrations, the infiltrated leaves were inoculated with TBSV crude sap inocula. The total RNAs were extracted and analyzed by northern blot assay as previously described [31].

### Confocal laser microscopy

The subcellular localization of AtRH30^ΔN/ΔC^ was observed in *N*. *benthamiana* plant epidermal cells after expression of an N-terminal fusion of AtRH30^ΔN/ΔC^ to GFP. In the experiments with TBSV and CNV, the transgenic *N*. *benthamiana* (expressing H2B fused to RFP, as a nuclear marker) leaves were infiltrated with agrobacteria carrying expression plasmids pGD-p33-BFP (OD_600_ 0.4), pGD-GFP-AtRH30^ΔN/ΔC^ (OD_600_ 0.4) and pGD-P19 (OD_600_ 0.2). The leaves were also co-infiltrated with agrobacterium to express CNV^20Kstop^ gRNA for CNV infections. For TBSV infections, the infiltrated leaves were inoculated with TBSV sap inocula 16 h post agroinfiltration. To observe the subcellular localization of GFP-AtRH30^ΔN/ΔC^ with cellular markers, the wild-type *N*. *benthamiana* leaves were agroinfiltrated to express p36-BFP and CoxIV-RFP (a mitochondria marker) in the case of CIRV infections or p33-BFP and RFP-SKL (peroxisome luminar marker) in the case of TBSV infections together with GFP-AtRH30^ΔN/ΔC^ and P19 (as a gene silencing suppressor). Approximately 2 days post-virus inoculations, imaging of infiltrated leaves was performed on an Olympus FV1200 confocal microscopy using 60X water-immersion objective equipped lasers as described [31]. BFP was excited by 405 nm laser. GFP was excited by 488 nm laser and RFP was excited by 543 nm laser. Images were obtained and merged using Olympus FLUOVIEW 1.5 software.

### BiFC assay

To detect the interaction between proteins in *N*. *benthamiana* plants, bimolecular fluorescence complementation (BiFC) assay was performed after protein expressions via agrobacterium infiltration. The leaves of wt *N*. *benthamiana* were infiltrated with agrobacteria carrying pGD-P19 (OD_600_ 0.2), pGD-RFP-SKL (OD_600_ 0.4) along with different combinations of plasmids as follows: pGD-nYFP-AtRH30 (OD_600_ 0.4) and pGD-T33-cYFP (OD_600_ 0.4) [62]; pGD-nYFP-AtRH30^ΔN/ΔC^ (OD_600_ 0.4) and pGD-T33-cYFP (OD_600_ 0.4); pGD-nYFP-MBP (OD_600_ 0.4, as a control) and pGD-T33-cYFP (OD_600_ 0.4); pGD-nYFP-AtRH20 (OD_600_ 0.4) and pGD-T33-cYFP (OD_600_ 0.4); pGD-nYFP-AtRH20^ΔN2-96^ (OD_600_ 0.4) and pGD-T33-cYFP (OD_600_ 0.4). After 16 h, infiltrated leaves of *N*. *benthamiana* plants were inoculated with TBSV crude sap inocula. Approximately 2 days post-virus inoculations, imaging of infiltrated leaves was performed as described above except YFP was excited by 514 nm laser.

### Analysis of TBSV replication with *in vitro* reconstituted TBSV replicase in yeast cell-free extract

The yeast cell-free extracts (CFE) that support *in vitro* TBSV replicase reconstitution were prepared from yeast strain BY4741 as described [18,54]. The *in vitro* reconstituted TBSV replicase assays were performed with the mixture of 2 μl of CFE, 0.5 μg DI-72 (+)repRNA, 0.2 μg affinity-purified MBP-p33 as well as MBP-p92^pol^ (both recombinant proteins were purified from E. *coli*), 5 μl buffer A (30 mM HEPES-KOH [pH 7.4], 150 mM potassium acetate, 5 mM magnesium acetate, 0.13 M sorbitol), 2 μl of 150 mM creatine phosphate, 0.2 μl of 10 mg/ml creatine kinase, 0.4 μl actinomycin D (5 mg/ml), 0.2 μl of 1 M dithiothreitol (DTT), 0.2 μl of RNase inhibitor, 2 μl a ribonucleotide (rNTP) mixture (10 mM of ATP, CTP, and GTP as well as 0.25 mM UTP) and 0.1 μl of [^32^P]UTP (NEB) in a total of 20 μl reaction volume. In addition, about 3.8 μM of purified recombinant proteins was also added in the reaction. The reaction was performed at 25°C for 3 h and then stopped by the addition of 5 volumes of 1% SDS and 5 mM EDTA, followed by phenol-chloroform extraction and RNA precipitation. Then the repRNA products and dsRNA intermediates were analyzed by electrophoresis in 0.5X Tris-borate-EDTA (TBE) buffer in a 5% polyacrylamide gel (PAGE) containing 8 M urea.

### Double stranded (ds)RNA separation assay

To study if the purified recombinant proteins could separate partial dsRNA duplexes, the dsRNA separation assay was performed as described [28]. First, the unlabeled single stranded (ss) TBSV (-)repRNA or TBSV (+)repRNA were synthesized by T7 polymerase-based *in vitro* transcription. On the other hand, ^32^P-labeled ss RI(-), RII(+),RIII(-) or RIV(+) RNAs was synthesized by T7-based *in vitro* transcription along with ^32^P-labeled UTP, respectively. To prepare partial dsRNA duplexes [i.e., RI(-)/ (+)repRNA, RII(+)/ (-)repRNA, RIII(-)/ (+)repRNA and RIV(+)/ (-)repRNA], 2 pmol of ^32^P-labeled ssRNA were annealed to 6 pmol of unlabeled repRNA in STE buffer (10 mM TRIS, [pH 8.0], 1 mM EDTA, and 100 mM NaCl) by slowly cooling down the samples (in a total volume of 20 μl) from 94°C to 25°C in 30 min in a PCR machine. An increasing amounts (0.95 μM, 1.9 μM, 3.8 μM and 7.6 μM or with 11.4 μM) of purified MBP fusion proteins or GST fusion proteins were added to the partial dsRNA duplexes in the RNA binding buffer (10 mM HEPES [pH7.4], 50 mM NaCl, 1 mM DTT, 1 mM EDTA, 5% Glycerol, 2.5 mM MgCl_2_) supplemented with 1 mM ATP, followed by incubation at 25°C for 25 min. The reaction mixtures were then treated with Proteinase K (2 μg/ per reaction) at 37°C for 20 min, followed by analysis with nondenaturing PAGE containing 5% polyacrylamide.

### Biotinylated RNA-protein interaction assay

To test if the RNA helicases and their mutants could interfere with viral template recognition by TBSV p33, we performed a biotinylated RNA-protein interaction assay as previously described [31] with a minor modification. The biotinylated RNA composed of the RII(+) of DI-72 was synthesized by *in vitro* T7 transcription supplemented with 1.5 mM of rATP, rCTP and rGTP as well as 0.75 mM rUTP along with 0.07 mM biotin16-UTP (Roche). Approximately 5.7 μM of recombinant MBP-RH30, MBP-RH30^ΔN/ΔC^, GST-RH20 and GST-RH20^ΔN2-96^, respectively, were incubated with 0.1 μg of biotinylated RNA, 0.1 μg of tRNA, 2 U RNase inhibitor, and 1 mM ATP or no ATP in the presence of biotin-RNA binding buffer (10 mM HEPES, 10% glycerol, 200 mM KCl, 5 mM MgCl_2_, 0.1% NP-40 [V/V], 1 mM DTT) in a total of 10 μL reaction volumes at 25°C for 15 min. Non-biotinylated RNA composed of the RII(+) of DI-72, MBP protein and GST protein were used as controls, respectively. After that, about 3.8 μM of the recombinant MBP-p33C was added to the reactions and incubated at 25°C for 15 min. The reaction mixtures were then incubated with 20 μL of streptavidin magnesphere paramagnetic particles (VWR) at room temperature for 20 min. The particles were captured by a magnetic stand and washed with biotin-RNA binding buffer for five times. The ribonucleoprotein complexes were eluted with 20 μL of SDS-PAGE loading dye containing β-mercaptoethanol by boiling for 15 min. The obtained samples were analyzed by western blot analysis with anti-MBP or anti-GST antibodies.

### Co-purification of RH30, RH20 and derivatives with the tombusvirus replication complex

For co-purification of RNA helicases with the membrane-bound TBSV replicase complex, the modified yeast strain BY4741, expressing an ADH promoter-driven His_6_-tagged p92 from the chromosome [51], was transformed with HpGBK-Cup1::Flag33/Gal1::DI-72 or HpGBK-Cup1::His33/Gal1::DI-72 (as controls) for the expression of Flag-tagged p33. The yeast cells were simultaneously co-transformed with different combinations of expression plasmids as follows: (i) LpESC-LEU-HisAtRH20 and UpYES-AtRH20ΔN2-96; (ii) LpESC-LEU-HisAtRH20 and UpYES-RH30ΔN/ΔC; (iii) LpESC-LEU-HisAtRH20 and UpYES2/NT empty vector (as controls); (iv) LpESC-LEU-HisAtRH30 and pYES-AtRH20; (v) LpESC-LEU empty vector (as controls) and pYES-AtRH20.

The co-purification assays were performed as previously described [31]. Briefly, the transformed yeast cells were grown in SC-ULH^-^ media supplemented with 2% glucose at 23°C for 16 h. After that, the cell cultures were transferred to SC-ULH^-^ media supplemented with 2% galactose for 24 h at 23°C, followed by the incubation with additional 50 μM CuSO_4_ for 6 h at 23°C. The obtained yeast cells were resuspended in a high-salt TG buffer (50 mM Tris-HCl [pH 7.5], 10% glycerol, 0.5 M NaCl, 10 mM KCl, 15 mM MgCl_2,_ 1% [V/V] yeast protease inhibitor cocktail [Ypic]) and were broken using a FastPrep Homogenizer (MP Biomedicals) and glass beads. Centrifugation at 500 g for 5 min at 4°C was performed to remove the cell debris. The membrane fraction containing the viral replicase complexes was collected by centrifugation at 35,000 g for 20 min at 4°C, followed by solubilization of membranes in a high-salt TG buffer containing 2% Triton X-100, 1% [V/V] Ypic for 3 h at 4°C. The supernatant of detergent-solubilized membranes was obtained by centrifugation at 35,000 g for 20 min at 4°C and then was incubated with anti-FLAG M2-agarose affinity resin (Sigma) in columns for 16 h at 4°C. The columns were washed with high-salt buffer for three times. For elution, the protein samples in the columns were incubated with SDS-PAGE loading dye at 85°C for 6 min and were collected by centrifugation at 500 g for 3 min. The collected samples were then boiled with β-mercaptoethanol for 20 min. Affinity-purified Flag-p33 was analyzed by western blot with anti-FLAG antibody, and co-purified 6xHis-tagged proteins was analyzed by western blot with anti-His antibody [31].

### Pull-down assay

To test direct binding between proteins, we performed an in vitro pull-down assay as previously described [63,64]. The cell lysates of *E*. *coli* strain BL21 (DE3) CodonPlus (Stratagene) expressing recombinant proteins, including MBP, MBP-AtRH30, MBP-AtRH30^ΔN/ΔC^, GST-his-T33C, GST, GST-AtRH20, GST-AtRH20^ΔN2-96^, and MBP-T33C were obtained as described above. The *E*. *coli* lysates expressing MBP or GST fusion proteins were separately incubated with amylose resin (NEB) or GST bind resin (EMD Millipore) for 4 h at 4°C, followed by washing with cold column buffer (20 mM HEPES [pH7.4], 25 mM NaCl, 1 mM EDTA [pH 8.0]) for four times. The amylose or GST bind resin was then incubated with E. *coli* lysates containing GST or MBP fusion proteins for 4 h at 4°C in the presence of 0.5% NP-40 and 0.1% [V/V] yeast protease inhibitor cocktail, followed by washing with cold column buffer containing 0.5% NP-40 for four times. The protein complexes bound to MBP resins were eluted with cold column buffer containing 0.36% [W/V] maltose and 1 mM DTT. For those bound to the GST bind resin were eluted with a column buffer containing 10 mM glutathione and 1 mM DTT in pH 7.5. The amounts of proteins were analyzed by western blot assay with anti-MBP or anti-His antibodies [31].

### Gel mobility shift assay (EMSA) and dsRNA strand-separation assay

The conditions for the EMSA experiments were described previously [16]. Briefly, the EMSA assay was performed with 0.1 pmol of ^32^P-labeled RNA probes along with different concentrations (0.4, 1.9, and 5.7 μM) of purified recombinant MBP-fusion proteins or MBP in the presence of RNA binding buffer (10 mM HEPES [pH7.4], 50 mM NaCl, 1 mM DTT, 1 mM EDTA, 5% Glycerol, 2.5 mM MgCl_2_), 2 U of RNase inhibitor, as well as 0.1 μg of tRNA in a total of 10 μl reaction volume. Two different amounts (2 and 4 pmol) of unlabeled RNAs together with 5.7 μM of either MBP-RH30 or MBP were used for template competition.

To study if purified proteins could unwind partial dsRNA duplex, the dsRNA strand-separation assay was performed as described [28]. Firstly, the unlabeled single-stranded DI-72 (-) or DI-72 (+) RNAs were synthesized via T7 polymerase- based in vitro transcription. The ^32^P-labeled single-stranded RI(-) or RII(+) RNAs were synthesized by T7-based in vitro transcription using ^32^P-labeled UTP. To prepare partial dsRNA duplexes, consisting of either RI(-)/DI-72 (+) or RII(+)/DI-72 (-) (see Fig 7E and 7F), 2 pmol of ^32^P -labeled RI(-) or RII(+) were annealed to 6 pmol of unlabeled DI-72(+) or DI-72 (-) in STE buffer (10 mM TRIS [pH 8.0], 1 mM EDTA, and 100mM NaCl) by slowly cooling down the samples (in a total volume of 20 μl) from 94°C to 25°C in 30 min. To test if the purified recombinant proteins could separate the partial dsRNA duplex, 1.9 and 5.7 μM purified MBP fusion proteins or MBP as a negative control were added separately to the partial dsRNA duplex in the RNA binding buffer (10 mM HEPES [pH7.4], 50 mM NaCl, 1 mM DTT, 1 mM EDTA, 5% Glycerol, 2.5 mM MgCl_2_) along with 1mM ATP, followed by incubation at 25°C for 25 min. The reaction mixtures were then treated with Proteinase K (2 μg/per reaction) at 37°C for 20 min, followed by loading onto 5% nondenaturing polyacrylamide gel with 200V for 1 h.

## Supporting information

S1 Text Materials and methods. (DOCX)

S1 Fig Comparison of amino acide sequences in the pro-viral RH20 and the antiviral RH30 DEAD-box helicases from *Arabidopsis*. The helicase core domains are boxed. Note that the N-terminal and C-terminal domains are the least conserved between the two helicases.
(DOCX)

S2 Fig Effect of expression of truncation mutants of the antiviral RH30 DEAD-box helicase on tombusvirus genomic RNA replication in *N. benthamiana* plant. (A) *N*. *benthamiana* plants transiently expressing truncated RH30 helicases were inoculated with TBSV. Top panel: Northern blot analyses of tombusvirus gRNA using a 3’ end specific probe shows the accumulation of gRNA and subgenomic RNAs in plants. Plants agroinfiltrated with the empty vector and expressing full-length RH30 were used as negative and positive controls, respectively. Bottom panel: northern blot shows 18S ribosomal RNA as a loading control. See further details in Fig 1. Each experiment was repeated at least three times. (B) Western blot analysis of the levels of HA-tagged-RH30 and derivatives with anti-HA antibody.
(DOCX)

S3 Fig Confocal microscopy shows the nuclear localization of RH30^ΔN^ helicase in plants. (A) Confocal microscopy images show that most of RH30^ΔN^ helicase is localized in the nucleus in the whole *N*. *benthamiana* plant and protoplasts marked with RFP-H2B. No CNV component was expressed. (B) Limited re-targeting of RH30^ΔN^ helicase into the VROs marked by the BFP-tagged p33 replication protein in *N*. *benthamiana* plants infected with CNV. (C-D) Partition of RH30 and RH30^ΔN/ΔC^ helicases between the cytosol and nucleus in the absence of viral components. The nucleus is marked by a histone protein (transgenic plants expressing nucleus marker RFP-H2B). Scale bars represent 10 μm. See further details in Fig 2.
(DOCX)

S1 Table The effect of deletions on the antiviral activity of RH30 DEAD-box helicase. (DOCX)

S2 Table The effect of deletions on the pro-viral activity of RH20 DEAD-box helicase. (DOCX)
