# Supplementary material for: Role reversal of functional identity in host factors: Dissecting features affecting pro-viral versus antiviral functions of cellular DEAD-box helicases in tombusvirus replication
Source: PLoS Pathog. 2020 Oct 9;16(10):e1008990. doi: 10.1371/journal.ppat.1008990 (PMC7577489; doi:10.1371/journal.ppat.1008990)
Supplement: S1 Text — (DOCX) [file ppat.1008990.s001.docx]

**Supplementary material:**

**S1 text. Materials and methods**

**Yeast strains and expression plasmids.** *Saccharomyces cerevisiae* strain BY4741 was obtained from Open Biosystems. To make deletion mutants within the N-terminal, helicase core or the C-terminal domains in AtRH30, the sequence of AtRH30^∆2-162^ (also named RH30^∆N^ for abbreviation), AtRH30∆163-591 (named RH30^∆Hel/∆C^), AtRH30∆2-162/∆547-591 (named RH30^∆N/∆C^) were PCR-amplified from plasmid pGD-AtRH30 [1] as follows: the sequence of RH30^∆N^ was PCR-amplified with primers #6709 (CCGCTCGAGATGCCGATGAAGA-TGTTCCAAGATGC) and #5753 (CGCGTCTAGATTACCAAGTCCTCTTTCCAC), followed by digestion with *Xho*I and *Xba*I. The digested product was ligated into *Xho*I/*Xba*I-digested pYES2/NT plasmid, resulting in pYES-AtRH30^∆N^. In addition, the sequence of RH30^∆Hel/∆C^ was PCR-amplified with primers #5754 (CGCGCTCGAGATGAGCTCGTATGATCGTAG) and #6708 (GCTCTAGATTAC-TTAGGAACATCACGACCTTCAAC), followed by the digestion with *Xho*I and *Xba*I. The digested product was ligated into *Xho*I/*Xba*I-digested pYES2/NT plasmid, resulting in pYES-AtRH30^∆Hel/∆C^. Moreover, the sequence of RH30^∆N/∆C^ was PCR-amplified with primers #6709 and #6710 (GCTCTAGATTATGATCGGACTAGTGCGGAGAGAG), followed by digestion with *Xho*I and *Xba*I. The digested product was ligated into *Xho*I/*Xba*I-digested pGD plasmid, resulting in pGD-AtRH30^∆N/∆C^.

To generate plasmids for expression of the N-terminally-fused Green Fluorescent Protein (GFP)-tagged AtRH30^∆N/∆C^, the sequence of AtRH30^∆N/∆C^ was PCR-amplified from plasmid pGD-AtRH30 with primers #6709 and #7838 (CCGCTCGAGTTATGATCGGACTAGTGCGGAGAGA), followed by digestion with *Xho*I. The digested product was then ligated into *Xho*I-digested pGDG plasmid [2], resulting pGD-GFP-AtRH30^∆N/∆C^, which was confirmed by PCR with primers #7198 (CATTTCTTTTAAAGCAAAAGC) and #7838 for the correct orientation of the ORF.

To clone deletion mutants within the N-terminal domain of AtRH20, first, a pGD expression plasmid fused with 3x repeats of HA-tag at an upstream position of the multiple cloning sites was created. The sequence of 3xHA was PCR-amplified from pESC-Ura-Vps34-3xHA [3] with primers #7391 (GAAGATCTATGGGTTACCCATACGATGTTC) and #7392 (CCGCTCGA-GCCAGGATCCAGCAGCGTAATCTGGAACGT), followed by the digestion with *Bgl*II and *Xho*I. The digested product was ligated into *BamH*I/*Xho*I-digested pGD plasmid, resulting in pGD-3HA-CY. The sequences of AtRH20 was PCR-amplified from plasmid pGD-AtRH20 [4] with #6509 (CGCGGATCCATGAGTCGCTACGATAGCCG) and #6972 (TGCTCTAGATCAGCTCCACCC-TCTTCTGCTC), followed by treatment with *BamH*I and *Xba*I. The digested product was then used for ligation into *BamH*I/*Xba*I-digested pGD-3HA-CY plasmid, resulting in pGD-3HA-AtRH20. The sequences of AtRH20^∆N2-36^, AtRH20^∆N2-58^ or AtRH20^∆N2-96^ were PCR-amplified from plasmid pGD-AtRH20 with primer pairs: #7051 (CCGCTCGAGATGTCTAGCAAAAAGGATA-ACGAT) and #6972, #7102 (CCGCTCGAGATGTTTGAGAAGAATTTTTATGTCGAGTCTCCCGC) and #6972 or #7393 (CCGCTCGAGATGCCTGTCAAGAGTTTTCGTGATGTTG) and #6972, respectively, followed by the digestion with *Xho*I and *Xba*I. These digested PCR products were used for ligations into XhoI/XbaI-digested pGD-3HA-CY plasmid, resulting in pGD-3HA-AtRH20^∆N2-36^, pGD-3HA-AtRH20^∆N2-58^ and pGD-3HA-AtRH20^∆N2-96^. In addition, the digested PCR products of AtRH20^∆N2-96^ were ligated into XhoI/XbaI-digested pYES2/NT plasmid, resulting in pYES-AtRH20^∆N2-96^.

To obtain the expression vectors for BiFC assay, an N-terminal nYFP expression vector carrying multiple restriction enzyme sites was generated. The sequence of nYFP was PCR-amplified from plasmid pGD-nYFP-MBP [5] with primers #5905 (GGAAGATCTATGG-TGAGCAAGGGCGAG) and #6069 (GCGCGGATCCGTCCTCGATGTTGTGGC), followed by digestion with *Bgl*II and *BamH*I. The digested fragment was ligated into *BamH*I-digested pGD plasmid, creating pGD-nYFP-CY. On the other hand, the previous *Xho*I/*Xba*I-digested AtRH30^∆N/∆C^ was used for ligation with *Xho*I/*Xba*I-digested pGD-nYFP-CY, resulting in pGD-nYFP-AtRH30^∆N/∆C^. Besides, the sequences of AtRH20 or AtRH20^∆N2-96^ were PCR-amplified from plasmid pGD-AtRH20 with primer pairs #6509 and #6972 or #7638 (CGGGATCCATGCCTGTCAA-GAGTTTTCGTGATGTTG) and #6972, respectively, followed by digestion with *BamH*I and *Xba*I. These digested products were used for ligations into *BamH*I/*Xba*I-digested pGD-nYFP-CY, resulting in pGD-nYFP-AtRH20 and pGD-nYFP-AtRH20^∆N2-96^.

To obtain the expression plasmids for MBP or GST fusion proteins, the sequence of RH30^∆N/∆C^ was PCR-amplified from plasmid pGD-AtRH30 with primers #7638 and #7639 (GCTCTAGAATGCCGATGAAGATGTTCCAAGATGC), followed by treatment with *Xba*I and *Xho*I. The digested product was ligated into *Xba*I/*Sal*I-digested pMAL-c2x vector, generating pMAL-AtRH30^∆N/∆C^. In addition, the previously *BamH*I/*Xho*I-digested products of AtRH20 or AtRH20^∆N2-96^ were used for ligation into pGEX-his-RE vector, producing pGEX-AtRH20 or pGEX-AtRH20^∆N2-96^.

To make chimeric constructs with AtRH20 and AtRH30, the N-terminal domains were swapped between AtRH20 and AtRH30. The sequence of AtRH20^∆Hel/∆C^ was PCR-amplified from plasmid pGD-AtRH20 with primers #1818 (CAGGCTCGAGATCATGAGTCGC-TACGATAGCCGGA) and #6849 (ATCTGGAAAGTTAGCATCTTGGAACATCTTGA-CAGGTTTTGGAATATCTTTGCCT). The sequence of AtRH30^∆N^ was PCR-amplified from pGD-AtRH30 with primers #6850 (GTTGAAGGCAAAGATATTCCAAAACCTGTCAAGA-TGTTCCAAGATGCTAACTTTC) and #5753. These two fragments were used as templates for PCR with primers #1818 and #5753, followed by digestion with *Xho*I and *Xba*I. The digested product was ligated into *Xho*I/*Xba*I-digested pGD-3HA-CY plasmid, resulting in pGD-3HA-RH233. On the other hand, the sequence of AtRH30^∆Hel/∆C^ was PCR-amplified from plasmid pGD-AtRH30 with primers #5754 and #6844 (ATCAGGAAAGCCAACATCACGAAAACTCTTCATC-GGCTTAGGAACATCACGACCT). The sequence of AtRH20^∆N^ was PCR-amplified from pGD-AtRH20 with primers #6845 (GTTGAAGGTCGTGATGTTCCTAAGCCGATGAAGAGTTTT-CGTGATGTTGGCTTTC) and #6972. These two fragments were used as templates together for PCR with primers #5754 and #6972, followed by digestion with *Xho*I and *Xba*I. The digested product was ligated into *Xho*I/*Xba*I-digested pGD-3HA-CY plasmid, resulting in pGD-3HA-RH322.

To clone the expression vector for co-purification assay in yeast, the sequence of AtRH20 containing a 6xHis-tag at the 5’-proximal end was PCR-amplified with primers #6493 (CGGGATCCATGCATCATCACCATCACCATAGTCGCTACGATAGCCGGAC) and #6491 (GGTTAATTAATCAGCTCCACCCTCTTC) from pGD-AtRH20. The PCR products were then treated with *BamH*I and *Pac*I, followed by ligation into *Bgl*II/*Pac*I-digested pESC-LEU plasmid, resulting in pESC-LEU-HisAtRH20. In addition, the PCR products of AtRH30 containing a 6xHistag at the 5’-proximal end was PCR-amplified with primers #6464 (CCGGCGGCCGCATG-CATCATCACCATCACCATAGCTCGTATGATCGTAGATTTGC) and #6465 (CCGTTAATTAATTACC-AAGTCCTCTTTCCACCGTGAG) from pGD-AtRH30. The PCR products were digested with *Not*I and *Pac*I, followed by ligation into *Not*I/*Pac*I-digested pESC-LEU plasmid, generating pESC-LEU-HisAtRH30. Moreover, the sequence of AtRH20 was PCR-amplified with primers #6509 and #6972 from pGD-AtRH20, followed by digestion with *BamH*I and *Xba*I. The digested products were ligated into *BamH*I/*Xba*I-digested pYES2/NT plasmid, producing pYES-AtRH20.

**Purification of recombinant proteins from E. *coli*.** Recombinant proteins GST-AtRH20, GST-AtRH20^∆N2-96^, GST, MBP-AtRH30, MBP-AtRH30^∆N/∆C^, MBP, MBP-p33, MBP-p92 were expressed in E. *coli* and affinity-purified as described [4]. Briefly, E. *coli* strain BL21 (DE3) CodonPlus (Stratagene) cells were transformed with expression plasmids to express the recombinant proteins. The obtained E. *coli* cells were cultured at 37°C for 16 h, followed by dilution of the culture to OD_600_ 0.2 with fresh media. The E. *coli* culture was then incubated at 37°C until reaching OD_600_ 1.0. The cultures were incubated in the presence of isopropyl-β-D-thiogalactopyranoside (IPTG) at 16°C for 8 h. The E. *coli* cells were then collected by centrifugation at 5,000 rpm at 4°C for 5 min, followed by resuspension with ice-cold column buffer (20 mM HEPES [pH7.4], 25 mM NaCl, 1 mM EDTA [pH 8.0]) containing 10 mM β-mercaptoethanol and 1 µg of RNase A in each of 4 ml E. *coli* suspensions. Sonication was performed on ice to get cell lysates, followed by centrifugation at 15,000 rpm at 4°C for 15 min. The obtained supernatants were incubated with GST binding resin (EMD Millipore) for GST fusion proteins or amylose resin (NEB) for MBP fusion proteins at 4°C for 2 h, respectively. The resins were then washed with ice-cold column buffer for four times. The recombinant proteins were eluted with column buffer containing 10 mM glutathione and 1 mM DTT in pH 7.5 for the GST fusion proteins or 0.36% [W/V] maltose and 1 mM DTT for the MBP fusion proteins.

**Quantification of blots and statistical analysis.** The quantification of TBSV gRNA was normalized to 18S rRNA level, followed by the quantification with ImageQuant 5.2. Data are presented as mean values and “±” was calculated as standard error. Data were then analyzed by GraphPad Prism 7 for statistical test. For the comparisons, One-way ANOVA with Dunnett’s test were used to calculate P value from data from at least triplicate experiments and these are stated in the corresponding figure legends. P< .05 was considered significance while P> .05 was considered no significance.

**References**

1. Wu CY, Nagy PD (2019) Blocking tombusvirus replication through the antiviral functions of DDX17-like RH30 DEAD-box helicase. PLoS Pathog 15: e1007771.

2. Goodin MM, Dietzgen RG, Schichnes D, Ruzin S, Jackson AO (2002) pGD vectors: versatile tools for the expression of green and red fluorescent protein fusions in agroinfiltrated plant leaves. Plant J 31: 375-383.

3. Feng Z, Xu K, Kovalev N, Nagy PD (2019) Recruitment of Vps34 PI3K and enrichment of PI3P phosphoinositide in the viral replication compartment is crucial for replication of a positive-strand RNA virus. PLoS Pathog 15: e1007530.

4. Kovalev N, Pogany J, Nagy PD (2012) A Co-Opted DEAD-Box RNA helicase enhances tombusvirus plus-strand synthesis. PLoS Pathog 8: e1002537.

5. Xu K, Nagy PD (2016) Enrichment of Phosphatidylethanolamine in Viral Replication Compartments via Co-opting the Endosomal Rab5 Small GTPase by a Positive-Strand RNA Virus. PLoS Biol 14: e2000128.
