# Supplementary figures and images for: Role reversal of functional identity in host factors: Dissecting features affecting pro-viral versus antiviral functions of cellular DEAD-box helicases in tombusvirus replication
Source: PLoS Pathog. 2020 Oct 9;16(10):e1008990. doi: 10.1371/journal.ppat.1008990 (PMC7577489; doi:10.1371/journal.ppat.1008990)

**S1 FIGURE**

**RH20: 501 aa**

**RH30: 592 aa**

Helicase core


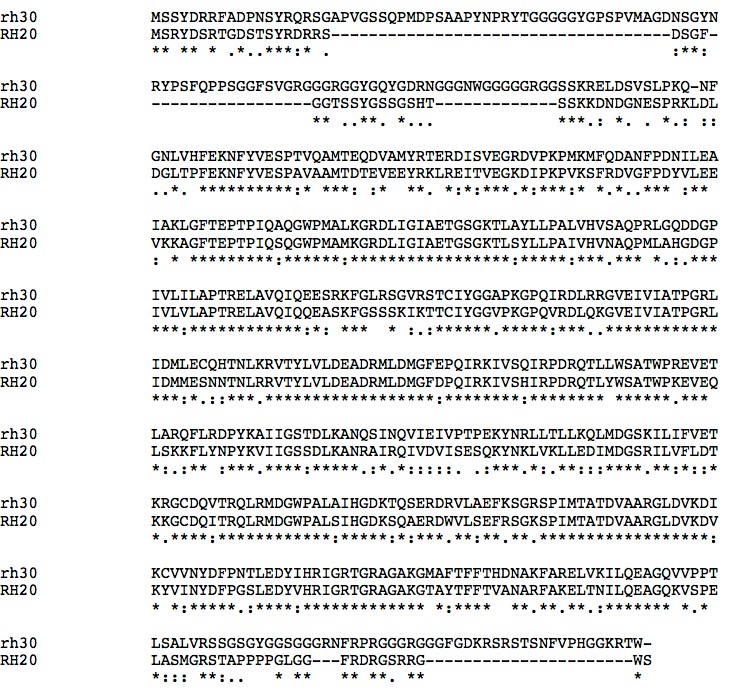

Supplement: S1 Fig — The helicase core domains are boxed. Note that the N-terminal and C-terminal domains are the least conserved between the two helicases. (DOCX) [file ppat.1008990.s002.docx]
