# Supplementary material for: Role reversal of functional identity in host factors: Dissecting features affecting pro-viral versus antiviral functions of cellular DEAD-box helicases in tombusvirus replication
Source: PLoS Pathog. 2020 Oct 9;16(10):e1008990. doi: 10.1371/journal.ppat.1008990 (PMC7577489; doi:10.1371/journal.ppat.1008990)
Supplement: S2 Fig — (A) N. benthamiana plants transiently expressing truncated RH30 helicases were inoculated with TBSV. Top panel: Northern blot analyses of tombusvirus gRNA using a 3’ end specific probe shows the accumulation of gRNA and subgenomic RNAs in plants. Plants agroinfiltrated with the empty vector and expressing full-length RH30 were used as negative and positive controls, respectively. Bottom panel: northern blot shows 18S ribosomal RNA as a loading control. See further details in Fig 1. Each experiment was repeated at least three times. (B) Western blot analysis of the levels of HA-tagged-RH30 and derivatives with anti-HA antibody. (DOCX) [file ppat.1008990.s003.docx]

**S2 FIGURE**


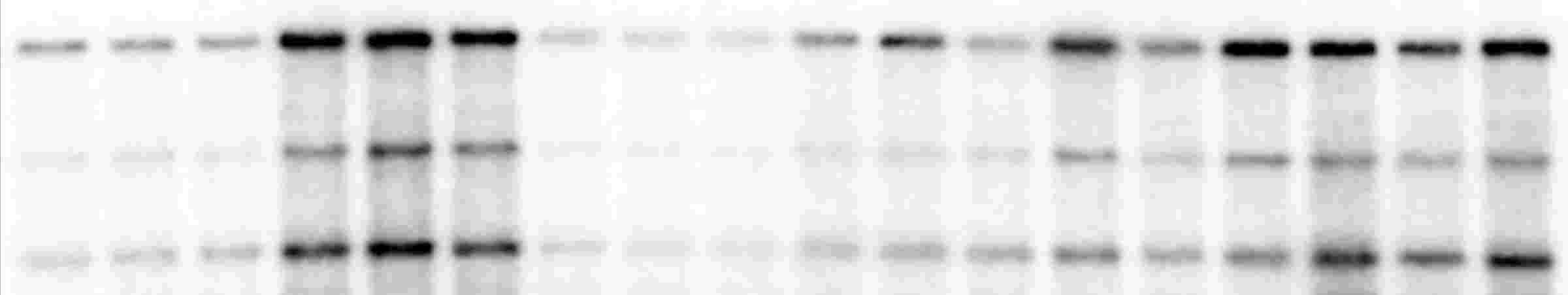

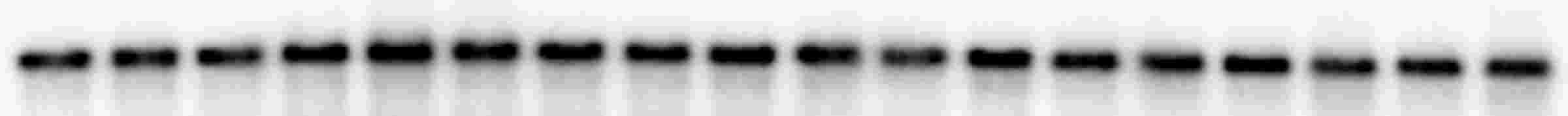

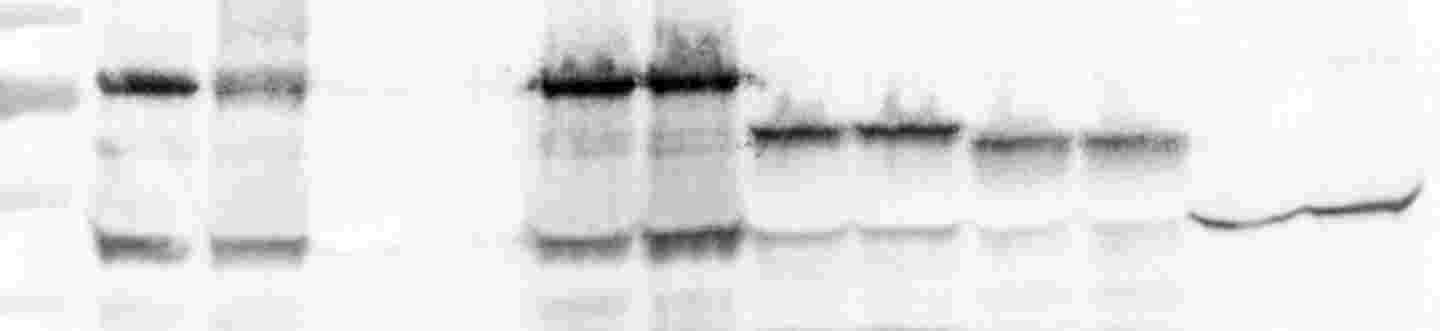

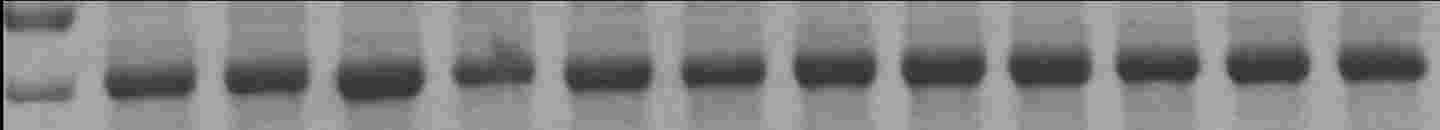


**gRNA**

**sgRNA1**

**sgRNA2**

**-**

**RH30**

∆Ν

**2-**

**17**

**rRNA**

**18**

**2 3 4 5 6 7 8 9 10 11 12 13 14 15 16 17**

**1**

**A.**

**TBSV RNA accumulation**

**+**

**17% gRNA**

**19 118**

**+**

**13 88**

**+**

**4 36**

**+**

**4 14**

**+**

**3 100**

**+**

**22**

**-**

**RH30**

**N2-162**

**B**

**RH30**

∆Ν

**2-**

**103**

**RH30**

∆Ν

**2-**

**124**

**RH30**

∆Ν

**2-**

**162**

**RH30**

**FL**

**MW**

**RH30**

∆Ν

**2-**

**17**

**RH30**

∆Ν

**2-**

**103**

**RH30**

∆Ν

**2-**

**124**

**RH30**

∆Ν

**2-**

**162**

**RH30**

**FL**

**total**

**70**

**55**
