# Supplementary material for: Role reversal of functional identity in host factors: Dissecting features affecting pro-viral versus antiviral functions of cellular DEAD-box helicases in tombusvirus replication
Source: PLoS Pathog. 2020 Oct 9;16(10):e1008990. doi: 10.1371/journal.ppat.1008990 (PMC7577489; doi:10.1371/journal.ppat.1008990)
Supplement: S3 Fig — (A) Confocal microscopy images show that most of RH30ΔN helicase is localized in the nucleus in the whole N. benthamiana plant and protoplasts marked with RFP-H2B. No CNV component was expressed. (B) Limited re-targeting of RH30ΔN helicase into the VROs marked by the BFP-tagged p33 replication protein in N. benthamiana plants infected with CNV. (C-D) Partition of RH30 and RH30ΔN/ΔC helicases between the cytosol and nucleus in the absence of viral components. The nucleus is marked by a histone protein (transgenic plants expressing nucleus marker RFP-H2B). Scale bars represent 10 μm. See further details in Fig 2. (DOCX) [file ppat.1008990.s004.docx]

**S3 FIGURE**

**A**

**p33-BFP**

**no CNV**

**no BFP**

**merged**

**GFP-RH30(**

∆

**N)**

**DIC**

**H2B-RFP**


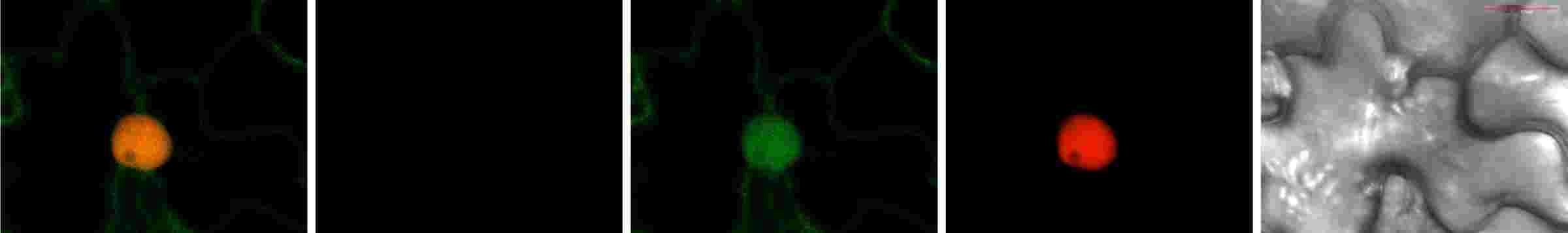

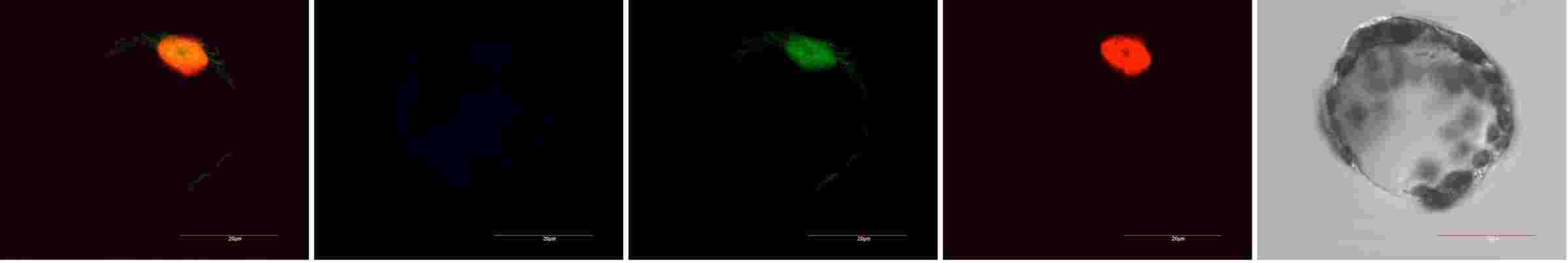


**protoplast**

**plant**

**merged**

**GFP-RH30(**

∆

**N)**

**DIC**

**ER-RFP**

**B**

**+**

**CNV**


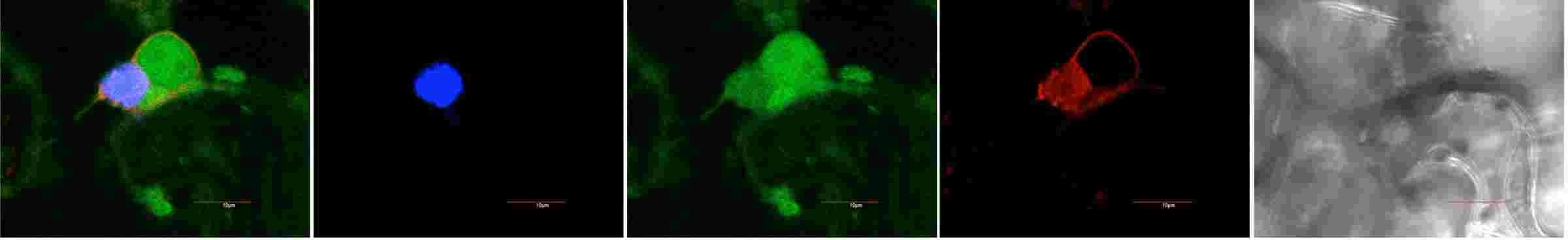


**RFP-RH30**

**merged**

**GFP-RH30**

**(**

∆

**N/**

∆

**C)**

**DIC**

**H2B-RFP**


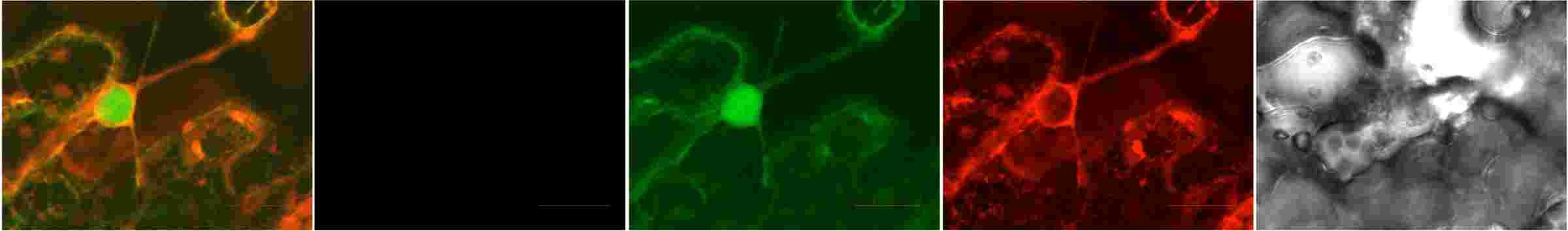

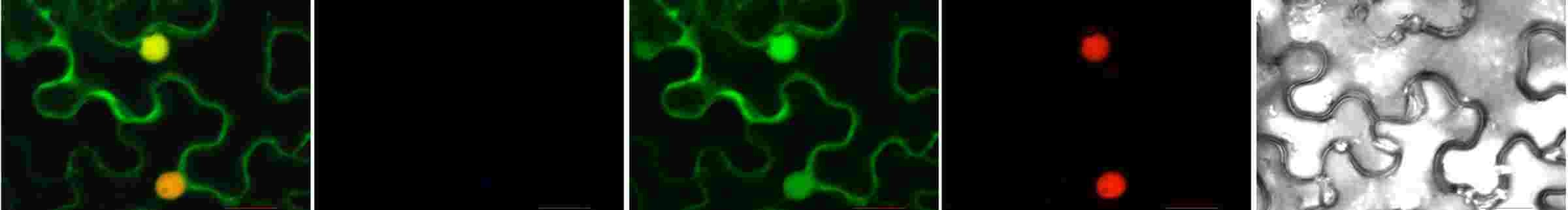

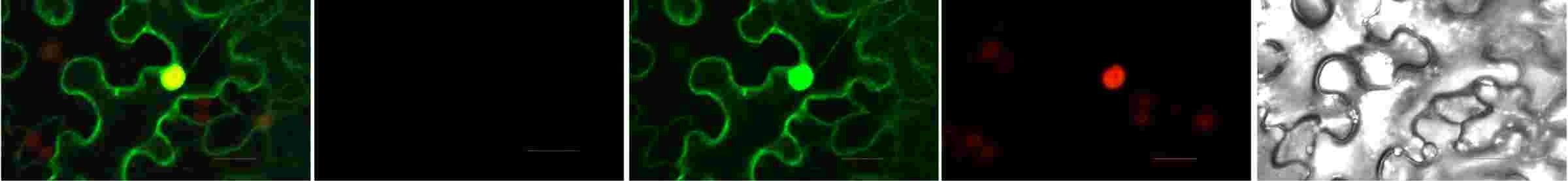


**no BFP**

**merged**

**DIC**

**ER-RFP**

**GFP-RH30**

**(**

∆

**N/**

∆

**C)**

**no CNV**

**no CNV**

**DIC**

**C**

**D**

**no BFP**


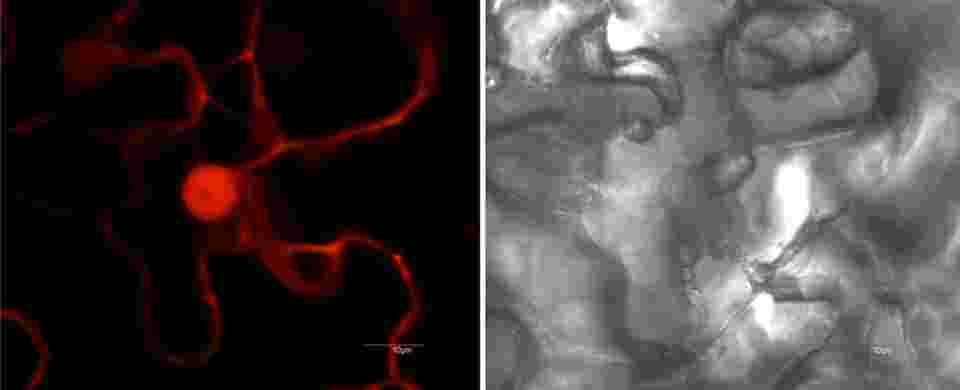


**no CNV**
