# Supplementary material for: Role reversal of functional identity in host factors: Dissecting features affecting pro-viral versus antiviral functions of cellular DEAD-box helicases in tombusvirus replication
Source: PLoS Pathog. 2020 Oct 9;16(10):e1008990. doi: 10.1371/journal.ppat.1008990 (PMC7577489; doi:10.1371/journal.ppat.1008990)
Supplement: S1 Table — (DOCX) [file ppat.1008990.s005.docx]

**S1 Table. The effect of deletions on the antiviral activity of RH30 DEAD-box helicase**

Name TBSV CIRV replication

RH30 FL 19+13 24+6

N-terminal mutants:

RH30^ΔN2-17^ 22+3 21+15

RH30^ΔN2-103^ 36+13 79+6

RH30^ΔN2-124^ 88+19 138+15

RH30^ΔN 2-162^ 118+17 105+15

Helicase core mutants:

RH30^F416L^ 111+31 101+8

RH30^ΔHel (Δ163-546)^ 92+11 77+30

C-terminal mutants:

RH30^ΔC547-592^ 25+7 5+4

Dual-mutants:

RH30^ΔHel/ΔC (Δ163-592)^ 93+1 99+25

**RH30^ΔN/ΔC^** ^(ΔN2-162/ΔC547-592)^ 200+27 190+10

___________________________________________________________

These helicase derivatives were transiently expressed in *N. benthamiana*. Northern blot was used to measure the viral accumulation level in the agroinfiltrated leaves (See S2 Figure as a representative). TBSV repRNA accumulation in yeasts expressing the 3xHA tag from a plasmid was taken as 100%. Standard error was calculated from three independent repeats. The mutant in bold is further characterized in this work.
