# Supplementary material for: Role reversal of functional identity in host factors: Dissecting features affecting pro-viral versus antiviral functions of cellular DEAD-box helicases in tombusvirus replication
Source: PLoS Pathog. 2020 Oct 9;16(10):e1008990. doi: 10.1371/journal.ppat.1008990 (PMC7577489; doi:10.1371/journal.ppat.1008990)
Supplement: S2 Table — (DOCX) [file ppat.1008990.s006.docx]

**S2 Table. The effect of deletions on the pro-viral activity of RH20 DEAD-box helicase**

Name TBSV CIRV replication

RH20 FL 161+30 175+10

N-terminal mutants:

RH20^ΔN2-36^ 114+42 40+7

RH20^ΔN2-58^ 30+16 45+11

**RH20^ΔN(ΔN2-96)^** 39+17 36+8

C-terminal mutants:

RH20^ΔC (ΔC 480-501)^ 166+20 ND

___________________________________________________________

These helicase derivatives were transiently expressed in *N. benthamiana*. Northern blot was used to measure the viral accumulation level in the agroinfiltrated leaves. See additional details in S1 Table legend. The mutant in bold is further characterized in this work.
